# Fungal extracellular vesicles mediate conserved cross-species communication and immunomodulation

**DOI:** 10.1128/mbio.03469-25

**Published:** 2026-01-30

**Authors:** Renan E. A. Piraine, Julia L. Froldi, Henrique T. Oliveira, Patrick W. Santos, Bianca T. M. Oliveira, Caroline P. Rezende, Lucas Alves Tavares, Gabriel E. S. Trentin, Lucas F. B. Nogueira, Arnaldo L. Colombo, Arturo Casadevall, Marcio L. Rodrigues, Fausto Almeida

**Affiliations:** 1 Department of Biochemistry and Immunology, Ribeirão Preto Medical School, University of São Paulo 54539, Ribeirão Preto, São Paulo, Brazil; 2 Department of Molecular Biology, Cancer Molecular Research Laboratory (LIMC)/ FAMERP 67736, São José do Rio Preto, São Paulo, Brazil; 3 Center for Virology Research and Department of Cell and Molecular Biology, Ribeirão Preto Medical School, University of São Paulo 54539, Ribeirão Preto, São Paulo, Brazil; 4 Special Mycology Laboratory, Division of Infectious Diseases, Federal University of São Paulo 28105, São Paulo, Brazil; 5 Antimicrobial Resistance Institute of São Paulo – ARIES, Federal University of São Paulo 28105, São Paulo, São Paulo, Brazil; 6 Centre for Medical Mycology in Latin America (CMM LATAM) Unit, São Paulo, São Paulo, Brazil; 7 Department of Molecular Microbiology and Immunology, Johns Hopkins School of Public Health 25802 , Baltimore, Maryland, USA; 8 Gene Expression Regulation Laboratory, Carlos Chagas Institute, FIOCRUZ 169688 , Curitiba, Paraná, Brazil; 9 Institute of Microbiology Paulo de Goes, Federal University of Rio de Janeiro (UFRJ) 28125 , Rio de Janeiro, Brazil; Texas Christian University, Fort Worth, Texas, USA

**Keywords:** virulence factors, pathogenic fungi, intraspecies, interspecies, immunostimulation, fungal communication

## Abstract

Extracellular vesicles (EVs) play crucial roles in fungal communication and host immune modulation, representing potential therapeutic targets for fungal infections. This study investigated the role of fungal EVs in both intra- and interspecies communication, focusing on their effects on virulence and immune responses. Co-incubation experiments were performed using EVs derived from *Candida albicans* and *Candidozyma auris* to assess interactions with *C. albicans* planktonic cells and biofilms, as well as *Cryptococcus neoformans* and *Cryptococcus gattii* EVs interacting with *C. neoformans* cultures. EVs were observed associating with recipient cell surfaces, suggesting subsequent internalization. Functional assays revealed that EV exposure led to increased expression of *CAP59, LAC1, URE1,* and *ERG11* genes, correlating with reduced antifungal susceptibility in both planktonic and biofilm forms. Additionally, EVs facilitated cross-species communication, enhancing biofilm adhesion and dispersion, which emphasizes their role in phenotypic modulation. Macrophages stimulated with fungal EVs exhibited receptor-specific gene expression changes (dependent on the EVs’ origin, including variation among species of the same genus), along with a pro-inflammatory phenotype marked by increased *iNOS* expression, enhanced TBK1/STAT1 production, and elevated levels of IL-1β, IL-6, and IL-8. Collectively, these findings underscore a critical role for fungal EVs in interspecies communication, biofilm regulation, and immune modulation, offering valuable insights into fungal pathogenicity mechanisms.

**IMPORTANCE:**

Currently, no vaccines exist to prevent fungal infections, underscoring the need for new therapies. As fungal diseases increase globally, understanding fungal biology is essential to identifying treatment targets. Fungi use extracellular vesicles (EVs) to communicate and evade immune responses. EVs mediate cell-cell communication, transporting proteins, polysaccharides, lipids, and nucleic acids, serving as “messages” exchanged within a fungal network. Understanding how these vesicles facilitate communication not only within a single species but also across different fungal species can shed light on their contribution to infection persistence and cross-species adaptability. Moreover, EVs may have a broader role in inter-kingdom signaling, influencing how fungi interact with host immune cells. The impact of fungal EVs on human innate immune responses remains a largely underexplored area, with significant gaps in our knowledge. This study aims to examine how fungal EVs affect immune responses and whether their signaling varies across species, potentially revealing new therapeutic targets.

## INTRODUCTION

In recent decades, fungi have emerged as significant human pathogens, posing a global threat to human health. This trend highlights the urgent need to address fungal infections, particularly with the rise of multidrug-resistant strains (1). The increase in fungal infections is closely associated with the rising number of immunocompromised individuals, as well as the fungi’s own evolving capabilities, such as enhanced pathogenicity and the ability to colonize new ecological niches (2–4). Fungal infections now affect over 1 billion people annually, with 2.55 million deaths reported worldwide, particularly among immunocompromised individuals (2, 5). Despite the considerable global health burden, the US Food and Drug Administration has yet to approve any vaccines for fungal infections (6). In 2022, the World Health Organization published its first-ever fungal priority pathogens list, which categorizes pathogens into critical, high, and moderate threats. Among the “critical importance” group are *Aspergillus fumigatus*, *Candida albicans*, *Candidozyma auris*, and *Cryptococcus neoformans*. These pathogens represent significant challenges due to their virulence and resistance to treatment (3, 7).

*Candida albicans* is one of the most prevalent *Candida* species associated with candidiasis in humans, causing nosocomial outbreaks with mortality rates exceeding 40% in some studies (1). In recent years, the multidrug-resistant fungus *Candidozyma auris* (formerly *Candida auris*) has emerged as a serious public health threat due to multiple outbreaks in immunocompromised individuals, repeatedly driven by its ability for long-term skin colonization, antimicrobial resistance, and high transmissibility (8). Effective control strategies, including the rational selection of antifungal therapies and rapid diagnostic tools, are crucial to prevent disease progression. Similarly, cryptococcosis, caused by *C. neoformans* and *C. gattii,* poses a comparable mortality risk due to severe pulmonary and central nervous system infections (1, 9). The ability of all of these fungi to establish infections is facilitated by several key virulence factors, such as thermotolerance, dimorphism, biofilm formation, capsule production, adhesins, and hydrolytic enzymes, all of which contribute to their pathogenicity and resistance to host defenses (3).

Fungal extracellular vesicles (EVs) play a critical role in pathogenesis, often described as “virulence bags” due to their role in transporting a variety of bioactive molecules, including proteins, lipids, nucleic acids, polysaccharides, toxins, and pigments. These components facilitate fungal dissemination and immune evasion within the host environment (10), functioning as carriers for molecules destined for the extracellular space (11). EVs are spherical structures enclosed by a bilayer membrane, categorized into small (<200 nm) and large (>200 nm) vesicles, following MISEV2023 (Minimal Information for Studies of Extracellular Vesicles) guidelines (12). Our group has shown that fungal EVs play a key role in facilitating communication among fungal cells within the same species (intraspecies interactions) (13). Additionally, these EVs also act as immunomodulators in host-pathogen interactions, as evidenced by *in vitro* studies involving immune cells (14–17).

Fungi can internalize EVs, triggering a variety of responses, including changes in gene expression. These responses are critical for fungal pathogens, influencing key processes such as antifungal tolerance, virulence, morphogenesis, and growth (13, 18). By coordinating fungal communities, EVs enhance their survival and adaptability within the host environment (19). EVs play a significant role in *C. albicans* biofilm formation and the yeast-to-hypha transition (13, 20, 21). However, further studies are needed to elucidate the roles of EVs in cross-species signaling and their impact on fungal biology during active infections.

Emerging studies are also investigating interactions between different *Candida* species mediated by EVs, prompted by epidemiological data suggesting that multispecies infections are becoming more common (22). In *Cryptococcus* species*,* EVs regulate key virulence mechanisms and enable long-distance signaling (23), with research on this topic spanning over the past 15 years (24, 25). Beyond their role in cell wall dynamics, cryptococcal EVs are critical for capsule biogenesis and maintenance, as they transport the major capsule component, glucuronoxylomannan (GXM), through a trans-cell wall export mechanism (11). Although coinfections involving different *Cryptococcus* species in humans are considered rare (26, 27), studying EV-mediated interspecies communication provides valuable insights into the conservation of cellular communication mechanisms across fungal species.

In addition to their role in cellular communication, EVs are potent stimulators of host immunity (28). Most research on the immunostimulatory effects of fungal EVs has been conducted *in vitro* using immune cells, primarily focusing on species from the *Candida* and *Cryptococcus* genera (28, 29). EVs from *C. albicans* were implicated in modulating both innate and adaptive immune responses, including the induction of nitric oxide (NO) production and the regulation of inflammatory response (30, 31). Key virulence factors in *C. albicans* EVs that contribute to host immune activation include phosphatidylserine, glucosylceramide, double-stranded DNA, and small RNAs (28, 31). In *Cryptococcus*, EVs also carry important virulence factors such as glucosylceramide and small RNAs. However, two other prominent factors, GXM and melanin, are known for their cytotoxic effects and ability to suppress phagocytosis, respectively (28, 32). While some studies report an anti-inflammatory response, characterized by increased IL-10 and TGF-β (transforming growth factor β) in macrophages stimulated with *C. neoformans* EVs (29, 33)*,* others have observed pro-inflammatory responses following stimulation with EVs from *C. neoformans* and *C. gatti* (32, 34).

In this study, we aimed to explore the role of fungal EVs in communication and immune modulation, isolating and characterizing EVs from *C. albicans, C. auris, C. neoformans,* and *C. gattii*. We hypothesized that EVs facilitate conserved intercellular signaling mechanisms among taxonomically divergent fungi. To address this hypothesis, we assessed EV-mediated intra- and interspecies communication by examining cellular uptake, functional consequences on planktonic growth and biofilm formation, alterations in antifungal susceptibility, and horizontal transfer of capsular polysaccharides. Additionally, we examined the immunomodulatory activity of these EVs on THP-1 human macrophage-like cells. This analysis focused on internalization, potential cytotoxic effects, the production of pro- and anti-inflammatory cytokines, mRNA expression, and protein levels of immune response-associated markers.

## RESULTS

### Fungal EVs as mediators in intra- and interspecies communication

**Fig 1:**
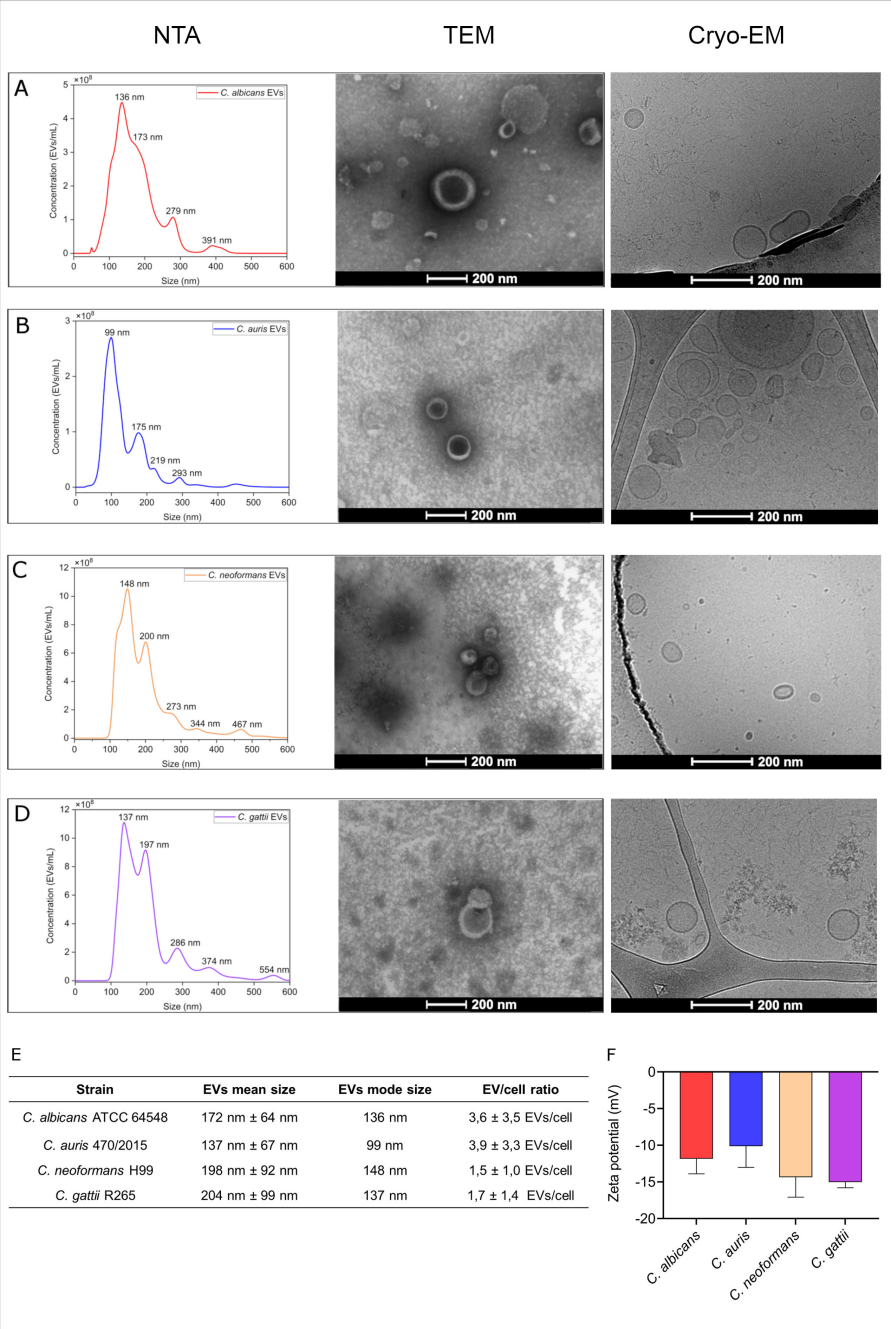
Characterization of fungal EVs. EVs isolated from *C. albicans* ATCC 64548 (**A**), *C. auris* 470/2015 (**B**), *C. neoformans* H99 (**C**), and *C. gattii* R265 (**D**) were analyzed by NTA, TEM, and transmission electron cryomicroscopy (cryo-EM). The data obtained from NTA are summarized in panel **E**. EV/cell ratio was calculated based on average cell concentration obtained after the first centrifugation step of the isolation protocol, which was compared to the EV total concentration detected by NTA. Zeta potential measurements of EVs were performed using dynamic light scattering and are shown in panel **F**. Statistical analysis was conducted using one-way ANOVA in GraphPad Prism 9.0. Error bars represent mean ± standard deviation.

The detailed characterization of EVs structure, cargo, and biological significance began around 2007 with the study of EVs from *C. neoformans* (29). Understanding the characteristics of EVs may reveal species-specific attributes that are critical for their function. Figure 1A through D presents the results of nanoparticle tracking analysis (NTA), transmission electron microscopy (TEM), and transmission electron cryomicroscopy (cryo-EM) for EVs isolated from cultures of *C. albicans* ATCC64548, *C. auris* clinical isolate 470/2015, *C. neoformans* H99, and *C. gattii* R265. Characterization of these EVs revealed a heterogeneous population in all samples, ranging from small EVs (sEVs) with a size of <200 nm to large EVs (lEVs) with >200 nm (summarized in Fig. 1E). The yield of EVs recovered after the isolation protocol (centrifugation, filtration, and ultracentrifugation) was quantified by the EV-to-fungal cell ratio, a method suggested for evaluating and comparing EV isolation from solid media-based cultures. Zeta potential, an important indicator of surface charge and stability, was also measured using dynamic light scattering. The zeta potential of fungal EVs ranged from −8 to −15 mV, indicating a stable colloidal suspension under physiological conditions (Fig. 1F). Other EVs used throughout this study were also characterized, and the results (zeta potential, NTA, and cryo-EM) are presented in the Supplementary Information file (Fig. S1A through D).

**Fig 2:**
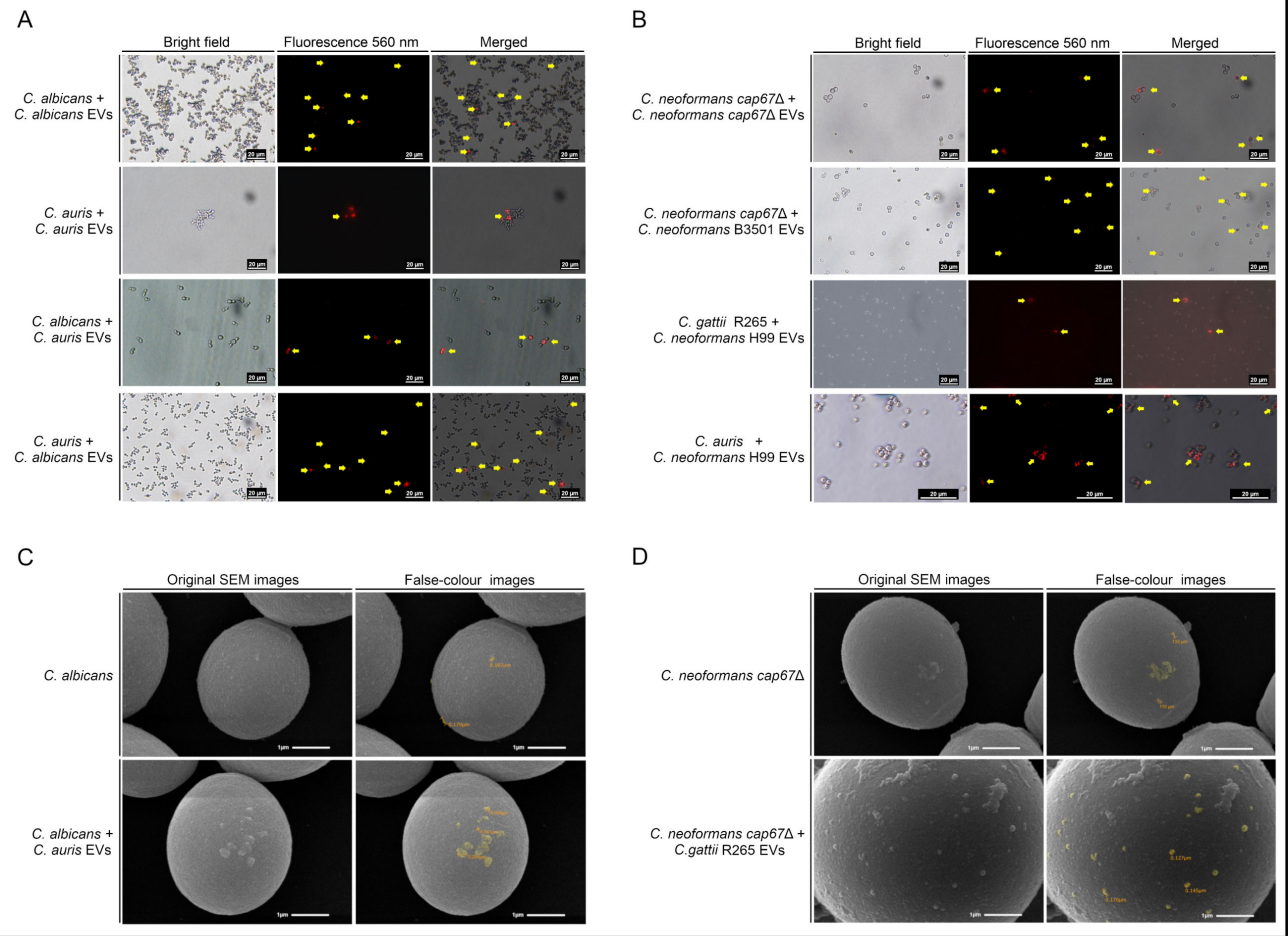
Interspecies cell surface association and internalization of fungal EVs. Fluorescence microscopy images suggest that fungal cells can recognize, interact with, and possibly internalize EVs produced by the same (control) or different species. Panel **A** shows interactions between yeasts and EVs from *C. albicans* and *C. auris,* while panel **B** depicts cells co-incubated with *C. neoformans* EVs. Yellow arrows indicate cells interacting with or internalizing EVs (400× magnification). SEM images in panels **C** and **D** show EV association with the fungal cell wall after co-incubation with *C. albicans* and *C. neoformans cap67*Δ, respectively, demonstrating interspecies EV-cell interactions. EVs are highlighted in yellow in the false-color images, which were manually edited using GIMP2 software and correspond to the original SEM images immediately to their left (magnification of 20,000×).

Our group previously demonstrated the role of fungal EVs in intraspecies communication (13). In this study, we explore the potential for EVs to serve as vehicles for interspecies communication, focusing on their ability to be “sent” and “received” as messages between fungi of different species. Using labeled EVs and fluorescence microscopy, we observed the recognition and possible internalization of EVs from both the same and different strains or species (Fig. 2A). Interactions were detected between *C. albicans, C. auris,* and *C. neoformans* (*cap67*Δ, an acapsular mutant strain) with their own EVs, and, more interestingly, between EVs and cells across genera, species, and strain backgrounds: *C. albicans* cells with *C. auris* EVs, *C. auris* cells with *C. albicans* EVs, *C. neoformans cap67*Δ cells with *C. neoformans* B3501 (mutant vs wild-type background) EVs, *C. gattii* R265 cells with *C. neoformans* H99 EVs, and even an interphylum interaction between *C. auris* cells and *C. neoformans* H99 EVs. Additionally, scanning electron microscopy (SEM) revealed an increase in vesicle-like structures adhered to the surface of recipient cells after incubations with EVs isolated from other species, supporting the hypothesis that EVs interact with the fungal cell wall and/or membranes before internalization (Fig. 2B).

EVs are frequently referred to as “virulence bags,” as they transport proteins and other biomolecules between cells (3). In our study, we explored the biological impact of EVs in interspecies communication, using *C. neoformans cap67*Δ as a model to better understand the role of EVs in the transmission of virulence factors. We chose the *C. neoformans cap67*Δ strain to avoid the capsule when exploring EV delivery, allowing for a more detailed examination of the cell surface without interference from capsular components that could hinder the visualization of EV incorporation. This approach was particularly valuable for investigating the transfer of GXM and other associated molecules from EVs derived from wild-type strains (B3501, H99, or R265) to determine whether the transfer of EV cargo is sufficient to restore the mutant phenotype. While *C. neoformans* var. *grubii* H99 is classified as serotype A, the *cap67*Δ mutant is a serotype D strain derived from strain B3501 (*C. neoformans* var. *neoformans*) (35). This mutant (*cap67*Δ) is also referred to as strain B4131 in the literature, and, as well as the *cap59*Δ strain (an acapsular mutant derived from H99), its phenotype can be restored by *CAP59* gene complementation (both strains show mutations in *CAP59*) (35, 36).

**Fig 3:**
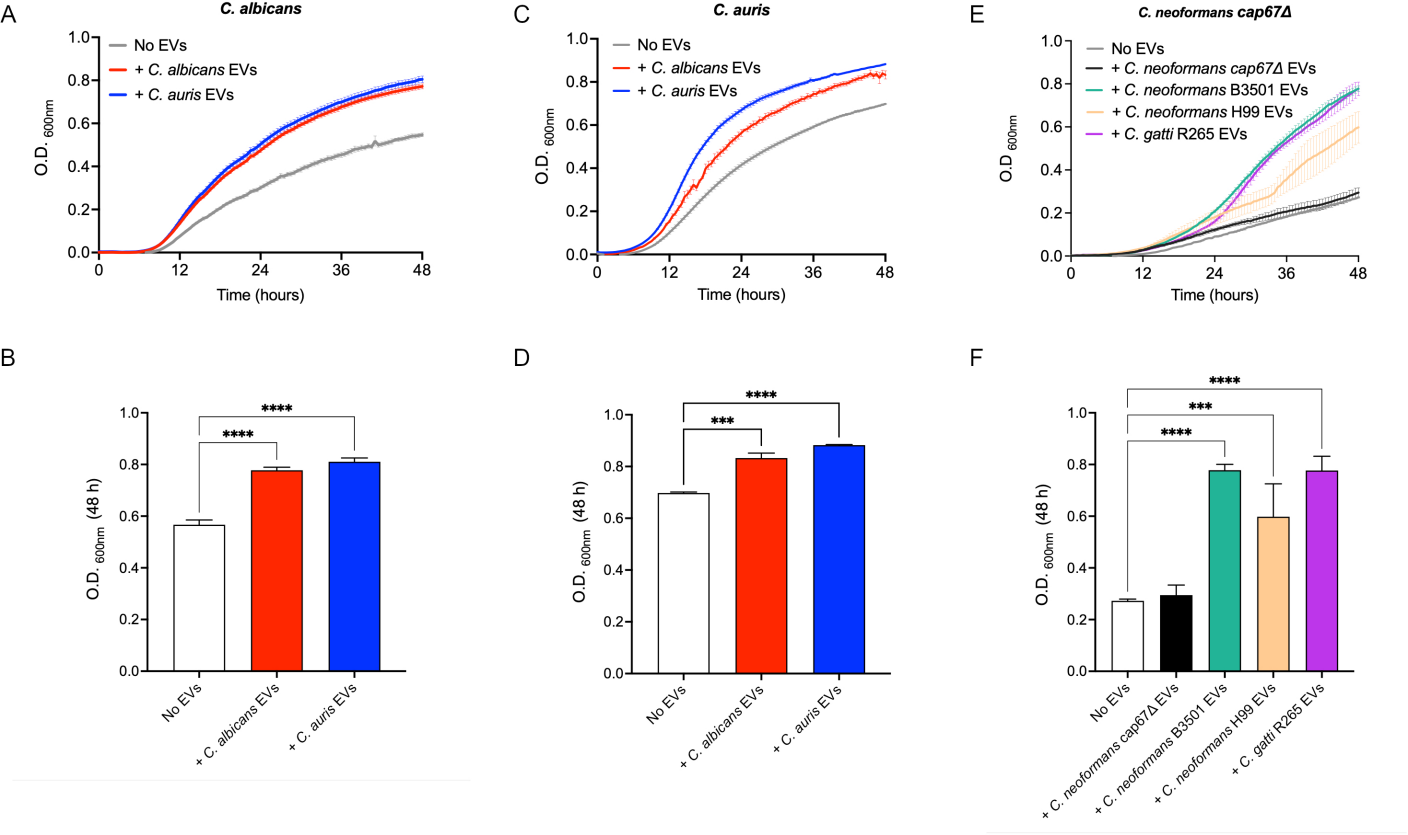
Growth curves and final OD_600nm_ (48 h) of *C. albicans, C. auris,* and *C. neoformans cap67*Δ after co-incubation with their own EVs or EVs from other species. Biological effect due to cross-species communication was first observed in the growth curves of yeasts incubated with EVs, indicating a direct impact on yeast physiology. (**A, B**) *C. albicans*; (**C, D**) *C. auris*; and (**E, F**): *C. neoformans cap67*Δ. Statistical analysis was performed using one-way ANOVA in GraphPad Prism 9.0, with significant differences indicated by the “*” symbol: ***P < 0.001 and ****P < 0.0001. Error bars represent means ± standard error of the mean .

Following the microscopy results, which revealed interactions between EVs and cells from both the same and different species in *Candida, Candidozyma,* and *Cryptococcus*, we next examined the biological effects of these interactions. We first evaluated the impact of EVs on yeast growth by monitoring optical density (OD_600nm_) over time, maintaining a 1:1,000 yeast-to-EV ratio in microplate wells. Both *C. albicans* (Fig. 3A and B) and *C. auris* (Fig. 3C and D) cultures displayed altered growth dynamics following EV addition, with a pronounced increase in absorbance regardless of the EV source or the yeast tested. In assays with *C. neoformans cap67*Δ, cells exposed to EVs from *C. neoformans* B3501, *C. neoformans* H99, and *C. gattii* R265 (but not to *cap67*Δ EVs) showed enhanced OD_600nm_ values, particularly after 24 h of incubation (Fig. 3E and F).

**Fig 4:**
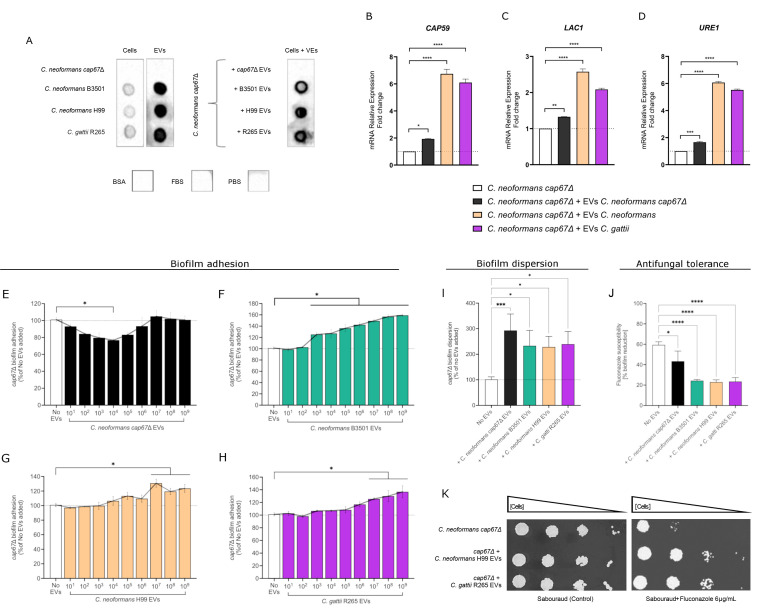
Biological impact of EVs derived from *C. neoformans* and *C. gattii* on planktonic cells and biofilms. (**A**) A dot blot using the anti-GXM monoclonal antibody was performed to detect the presence of capsular content in *Cryptococcus* EVs. EVs were applied (10 μL) at a concentration of 1 × 10^10^ EVs/mL. A gray/black dot confirmed the presence of GXM molecules in EVs from *C. neoformans* and *C. gattii* wild-type strains, and their absence in EVs from *C. neoformans cap67*Δ knockout. Bovine serum albumin (BSA) 5%, FBS 10%, and PBS were used as negative controls, while *C. neoformans* B3501, *C. neoformans* H99, and *C. gattii* R265 cultures were used as positive controls. The dot blot analysis was also performed using *C. neoformans cap67*Δ planktonic cells co-incubated with their own previously isolated EVs, and with EVs from *Cryptococcus* wild-type strains, to observe the transfer or induction of capsular material production mediated by EVs. mRNA relative expression levels of *CAP59* (**B**)*, LAC1* (**C**)*,* and *URE1* (**D**) were measured by qPCR and compared to the control (no EV treatment). The dotted lines represent the basal expression in *C. neoformans cap67*Δ cultures. (**E–H**) EVs from *C. neoformans cap67*Δ, *C. neoformans* B3501, *C. neoformans* H99, and *C. gattii* R265, at concentrations ranging from 10^1^ to 10^9^ EVs/well were added to biofilms of *C. neoformans cap67*Δ. Biofilm adhesion was evaluated at each EV concentration, and the concentration with the most pronounced biological effect was selected for further analysis of biofilm dispersion (**I**) and susceptibility to fluconazole (**J**). The dotted lines represent the biofilm formed by non-treated *C. neoformans cap67*Δ (no EVs added). Statistical analysis was performed using one-way ANOVA in GraphPad Prism 9.0, with significant differences indicated by the “*” symbol: *P < 0.05, **P < 0.01, ***P < 0.001, and ****P < 0.0001. Error bars represent means ± standard deviation. (**K**) Spot tests were conducted with dilutions of *C. neoformans cap67*Δ cultures, with or without EVs (control), to assess the effect on antifungal tolerance (fluconazole) in planktonic cells.

We first confirmed the presence of GXM in the EVs of *C. neoformans* B3501, H99, and *C. gattii* R265 by dot blot analysis (Fig. 4A), demonstrating a strong GXM signal at the tested concentration (10^8^ EVs). In contrast, EVs derived from the *cap67*Δ mutant strain were not recognized by antibodies specific to GXM. Co-incubation of EVs from wild-type strains (B3501, H99, and R265) with *cap67*Δ mutant cells revealed that GXM was incorporated into the mutant’s surface, indicating the uptake of a significant amount of the polysaccharide. Even after successive washes of the *cap67*Δ cells co-incubated with EVs, GXM detection was maintained in the dot blot assay, at levels visually comparable to those observed for the wild-type strains. Since GXM adsorption yielded similar results following co-incubation with EVs from both *C. neoformans* and *C. gattii*, these findings suggest that the transfer of the molecule may occur independently of the EV source. SEM images (Fig. 2B) further demonstrated that the acapsular mutant did not fully acquire a capsular structure after 24 h of co-incubation, in contrast to the complete phenotype reversal seen in the wild-type strain. This was further confirmed by India ink staining, where no significant differences were observed between cells that received EVs and the controls (no EVs added) (Fig. S2).

Next, we examined the effect of adding EVs to *C. neoformans cap67*Δ planktonic cells to assess their impact on virulence factor expression at the molecular level. Specifically, we selected three genes*—CAP59*, *LAC1*, and *URE1*—and measured their transcription by qPCR. Our results showed increased expression of these genes when mutant cells were incubated with EVs from *C. neoformans* and *C. gattii* (Fig. 4B through D). This response was observed in both intra- and interspecies interactions. Even *C. neoformans cap67*Δ EVs (Fig. S1C, Supplementary Information for EVs characterization) induced a modest increase in mRNA expression of these genes, albeit at lower levels. Overall, the addition of EVs led to an upregulation of all tested genes, suggesting that EVs directly contribute to the induction of virulence factor production.

The characteristics of *C. neoformans cap67*Δ biofilms were also altered following exposure to EVs. While the addition of *cap67*Δ EVs in a range of 10^1^ to 10^6^ EVs/well showed a trend toward reduced biofilm adhesion (with statistical significance observed only at 10^4^ EVs/well) (Fig. 4E), incubation of *cap67*Δ cells with *C. neoformans* B3501 EVs induced a clear dose-dependent effect (Fig. 4F), enhancing adhesion even at lower concentrations of EVs (10^3^ EVs/well) and continuing to increase up to the highest concentration tested (10^9^ EVs/well). When EVs from *C. neoformans* H99 and *C. gattii* R265 were used, significant increases in the number of adhered cells were observed at concentrations ranging from 10^7^ to 10^9^ EVs/well during the adhesion phase (Fig. 4G through H). The highest concentration of EVs was also tested in the biofilm dispersion assay, where it was shown that EVs influence the dispersion phase. However, no statistically significant differences were observed in the levels of dispersion between EVs from the same or different strains (Fig. 4I).

The impact of EVs on antifungal susceptibility was assessed, suggesting that biofilms incubated with EVs from the wild-type strains of *C. neoformans* and *C. gattii* exhibited increased tolerance to antifungals (Fig. 4J). Fluconazole (an azole) was chosen to evaluate EV-mediated effects on an antifungal target, the ergosterol biosynthesis. The effect on virulence factors was further confirmed by a spot test, where planktonic cells co-incubated with EVs from *C. neoformans* and *C. gattii* showed reduced antifungal susceptibility on agar plates containing various concentrations of fluconazole (Fig. 3K and Fig. S3).

**Fig 5:**
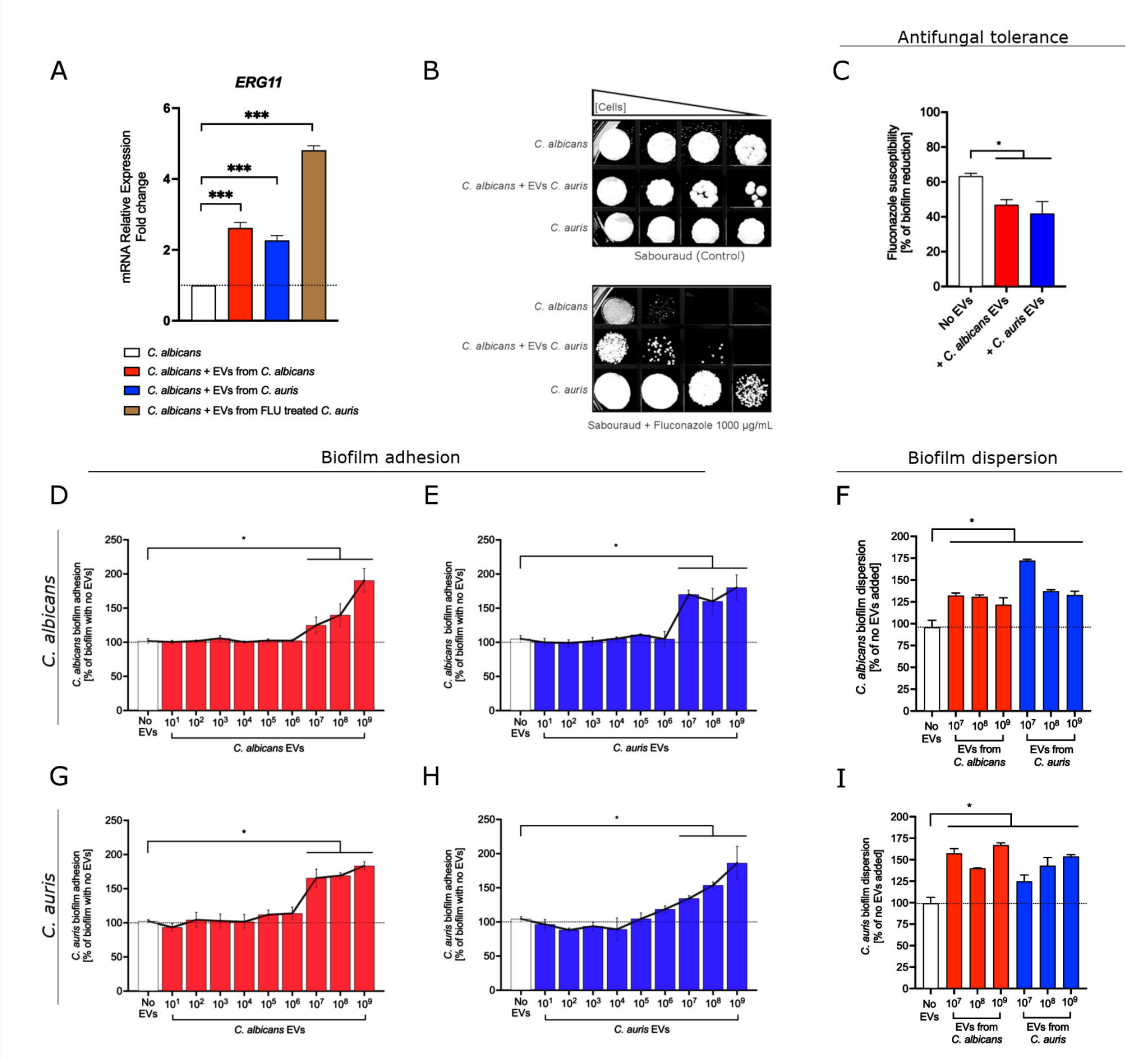
Biological impact of EVs from *C. albicans* and *C. auris* on planktonic cells and biofilms. (**A**) Planktonic *C. albicans* cells were incubated with EVs isolated from *C. albicans*, *C. auris*, or *C. auris* treated with fluconazole. *ERG11* mRNA relative expression was measured by qPCR and compared to the control (no EV treatment). The dotted line represents the basal expression in *C. albicans* cultures. (**B**) Spot tests were conducted with dilutions of *C. albicans* cultures that were treated with or without EVs (control strains: *C. albicans* and *C. auris*) on Sabouraud agar plates with or without fluconazole. (**C**) Fluconazole susceptibility in *C. albicans* (a fluconazole-susceptible strain) biofilms was tested following the addition of *C. albicans* EVs or *C. auris* EVs (10^9^ EVs/mL). (**D–F**) Different concentrations of EVs from *C. albicans* and *C. auris* were added during the adhesion and dispersion phases of *C. albicans* biofilms. (**G–I**) Different concentrations of EVs from *C. albicans* and *C. auris* were added during the adhesion and dispersion phases of *C. auris* biofilms. For all biofilm assays, the dotted lines represent results for biofilms with no EV addition. Statistical analysis was performed using one-way ANOVA in GraphPad Prism 9.0, with significant differences indicated by the “*” symbol: *P < 0.05 and ***P < 0.001. Error bars represent means ± standard deviation.

The biological impact of fungal EVs on interspecies communication was further demonstrated in both planktonic cells and biofilms of *Candida* and *Candidozyma* yeasts. In planktonic *C. albicans* cells, EVs isolated from both *C. albicans* and *C. auris* induced an increase in mRNA expression of *ERG11*, a gene that is upregulated in azole-resistant isolates. EVs isolated from *C. auris* cultured in fluconazole-containing plates (see Fig. S1B, Supplementary Information for *C. auris* NTA result) also stimulated elevated *ERG11* expression in planktonic *C. albicans*. Notably, qPCR analysis revealed that the increase in *ERG11* expression was approximately twofold higher than that observed with EVs from *C. albicans* or untreated *C. auris* EVs (Fig. 5A). Additionally, spot tests demonstrated a difference in fluconazole susceptibility between planktonic *C. albicans* cells incubated with EVs from *C. auris*, in which, at high fluconazole concentrations, *C. albicans* cells exposed to *C. auris* EVs exhibited a more tolerant phenotype (Fig. 5B). As seen in the assay with planktonic cells, exposure to EVs also altered azole tolerance in *C. albicans* biofilms. Fluconazole treatment resulted in an approximately 62% reduction in cell viability of biofilm mass (control); however, biofilms treated with *C. albicans* EVs showed a reduction of approximately 47%, and those treated with *C. auris* EVs exhibited a reduction of 42%, suggesting a decreased susceptibility to fluconazole in the presence of EVs (Fig. 5C).

The cross-communication between *C. albicans* and *C. auris* through EVs was also evaluated using assays of biofilm adhesion and dispersion. In biofilms of both *C. albicans* and *C. auris*, fungal cell/EV ratios ranging from 1:10 to 1:1,000 (representing 10^7^ to 10^9^ EVs/well) resulted in significantly increased biofilm adhesion compared to the control (no EVs added). No differences were observed when EV concentrations were ≤10^6^ EVs/well (Fig. 5D, E, G, and H). The EV dose range that produced effects on biofilms resembled that observed in *Cryptococcus* assays, supporting a possible role for EV concentration in interspecies communication among these yeasts. The EV concentrations that enhanced biofilm adhesion were selected for further investigation of their impact on biofilm dispersion. In these assays, biofilms treated with EVs, whether from *C. albicans* or *C. auris*, displayed higher levels of dispersed cells compared to the control (Fig. 5F and I), indicating that even phylogenetically distant pathogenic fungi can engage in cross-communication that promotes biofilm dispersal.

### Fungal EVs as molecules with immunomodulatory properties

**Fig 6:**
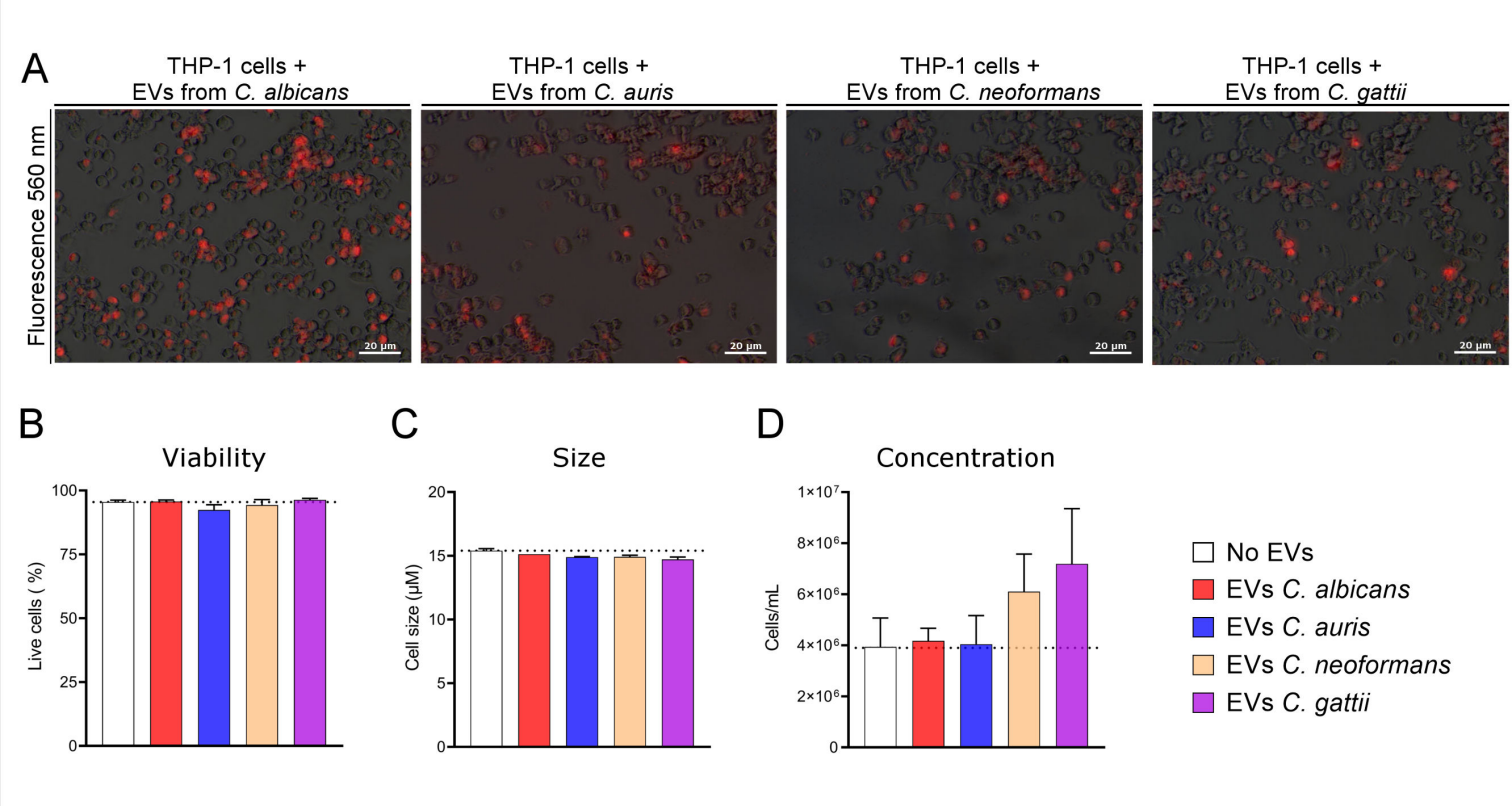
Interaction between fungal EVs and THP-1 macrophages, and their impact on cellular properties. (**A**) The internalization of EVs by THP-1 cells was observed through fluorescence microscopy, demonstrating successful recognition of and interaction with human macrophages. Micrographs from brightfield and under fluorescence (560 nm) were merged using GIMP 2 software. The biological effects on THP-1 cells following EV interaction were assessed by measuring the number of live cells (**B**), cell size (**C**), and cell concentration (**D**). Dotted lines indicate the data observed for untreated cells (control).

One of the critical factors in host-pathogen interactions during infection is communication via EVs (34). Fungal EVs play a pivotal role in fungal pathogenicity, as they transfer virulence factors with immunomodulatory properties. These factors are recognized as pathogen-associated molecular patterns (PAMPs), thereby activating the innate immune response (3, 35). Similar to the assays performed with fungal cells, we labeled fungal EVs with a fluorescent dye to observe their incorporation by immune system cells, specifically human macrophage-like cell cultures (THP-1 cells). Through fluorescence microscopy, we detected labeled EVs overlapping with regions where THP-1 cells were located (Fig. 6A), suggesting an association with the macrophage-like cell surface and potential internalization. Since the cytotoxic effects of fungal EVs vary depending on the study and experimental conditions, we further investigated their impact on cellular properties during co-incubation of THP-1 cells with EVs from *C. neoformans* H99, *C. gattii* R265, *C. albicans,* and *C. auris*. The assay revealed no significant differences in human macrophage-like cells cultured *in vitro* concerning cell size, viability, or concentration following exposure to fungal EVs (Fig. 6B through D).

**Fig 7:**
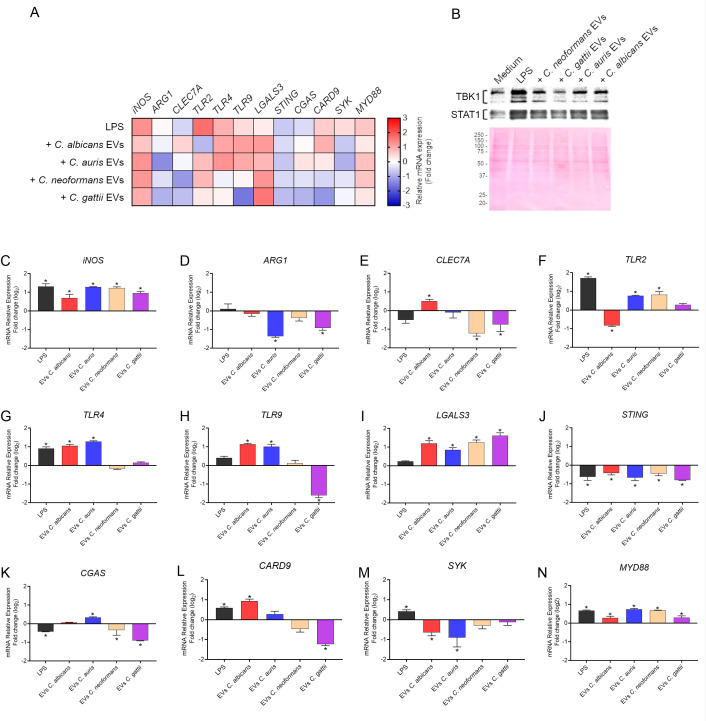
mRNA and protein levels of immune response markers in THP-1 cells stimulated with fungal EVs. This figure presents the relative expression levels of macrophage polarization markers (*iNOS*, *ARG1*), receptors (*TLR2*, *TLR4*, *TLR9*, *CLEC7A*, *LGALS3*), and proteins involved in key immune signaling cascades (*STING*, *CGAS*, *CARD9*, *SYK*, *MYD88*). qPCR results were normalized to the housekeeping β-actin gene (*ACT1*) and calculated using the 2^-ΔΔCT^ method. The heatmap presented in (**A**) summarizes the results observed for relative mRNA transcription. Fold change for relative mRNA transcription is presented in the graphs (**C–N**) as log_2_ to facilitate up- and downregulation comparisons. Statistical analysis was performed using one-way ANOVA in GraphPad Prism 9.0, with significant differences indicated by the “*” symbol: *P < 0.05. Error bars represent means ± standard deviation. Western blots performed to detect STAT1 and TBK1 proteins are presented in panel **B**. The Ponceau S-stained membrane is shown to demonstrate equal loading of total protein among samples, with protein bands displayed within the 20–250 kDa molecular mass range.

The stimulation of THP-1 macrophage-like cells by fungal EVs was analyzed using qPCR, targeting the mRNA transcription of polarization markers, receptors, and proteins involved in immune response pathways associated with combating pathogenic fungi (Fig. 7A and C through M). All fungal EVs induced an upregulation of *iNOS* (nitric oxide synthase) and either a basal or downregulated expression of *ARG1* (arginase 1) (*P* < 0.05). This response suggests the polarization of macrophages toward the M1 phenotype. We also determined the production of two different proteins involved in cell signaling during innate immune response (Fig. 7B). Stimulation with fungal EVs resulted in a slight but notable increase in STAT1 (signal transducer and activator of transcription 1), in agreement with the mRNA expression results suggesting polarization toward M1 macrophages. Enhanced production of TBK1 (TANK-binding kinase 1) was also detected after stimulation with fungal EVs, consistent with its role in promoting pro-inflammatory cytokine expression in immune cells.

*Candida* and *Candidozyma* EVs stimulated human macrophages to upregulate the mRNA expression of *TLR4* and *TLR9*. Among these, only *C. albicans* induced significant levels (*P* < 0.05) of *CLEC7A* expression, while *C. auris* specifically stimulated *TLR2*. In contrast, *Cryptococcus* EVs generally showed either basal mRNA expression (not statistically different) or downregulation of the evaluated receptors. For instance, *CLEC7A* was downregulated in response to EVs from both cryptococcal species, and *TLR9* was downregulated following stimulation with EVs from *C. gattii*. Notably, *TLR2* was the only receptor upregulated by *Cryptococcus* EVs, with its expression significantly enhanced in response to *C. neoformans* EVs. Additionally, *LGALS3*, the gene encoding Galectin-3, a protein that binds beta-galactosides and exhibits antimicrobial activity, was consistently upregulated in THP-1 cells across all EVs tested.

The immunomodulatory effects of fungal EVs exhibited a pattern of *STING* downregulation. Specifically, *C. albicans* and *C. auris* EVs downregulated *SYK* expression, while *Cryptococcus* EVs led to the expression of *CGAS*. Regarding *CARD9*, EVs from *C. neoformans* caused a significant downregulation of its expression, whereas incubation with *C. albicans* EVs resulted in increased mRNA expression in THP-1 cell cultures. *MYD88*, a gene encoding an adapter protein widely studied in immune responses, was upregulated in response to stimulation by all fungal EV stimuli, with the most pronounced effects observed for EVs derived from *C. auris* and *C. neoformans*. Although the mRNA expression data are interesting and suggestive of a potential modulation of recognition and signaling pathways following exposure to fungal EVs, they represent only part of the information necessary to establish the immunomodulatory effect, still requiring deeper protein-level analyses to validate this EV-mediated activity.

**Fig 8:**
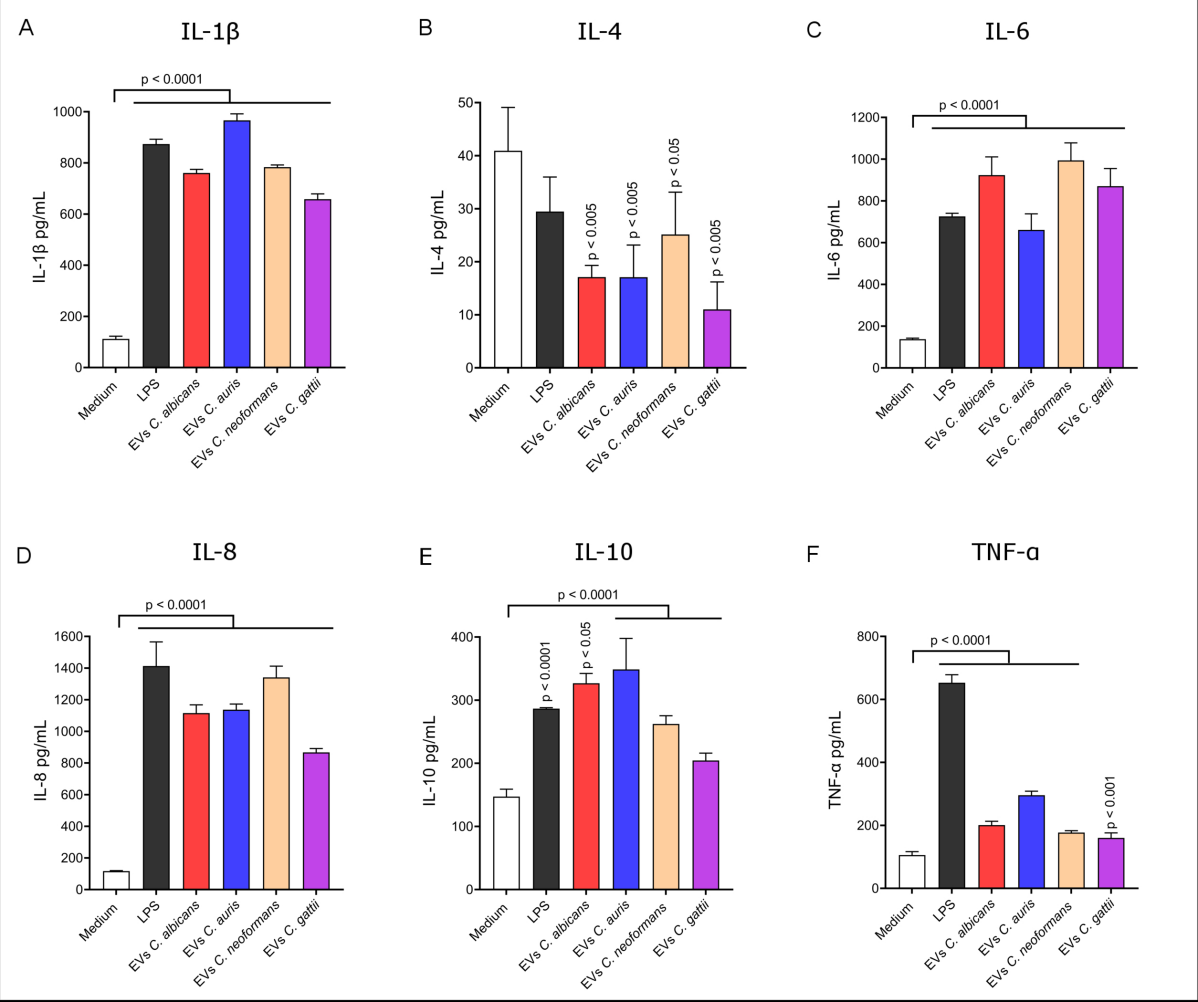
Cytokine production by THP-1 macrophages following stimulation with fungal EVs. THP-1 cells were cultured either with medium only (control) or with EVs derived from *C. albicans*, *C. auris*, *C. neoformans*, and *C. gattii*. The production of IL-1β (**A**), IL-4 (**B**), IL-6 (**C**), IL-8 (**D**), IL-10 (**E**), and TNF-α (**F**) was quantified by ELISA. Statistical analysis was performed using GraphPad Prism 9.0 with a one-way ANOVA test. Significant differences are indicated by *P*-values above the bars, compared to the control (medium only). Error bars represent the means ± standard deviation.

The quantification of pro- and anti-inflammatory cytokines in the supernatant of macrophage cultures performed by ELISA allowed the characterization of the immune response profile mediated in THP-1 cells stimulated by fungal EVs. Pro-inflammatory cytokines IL-1β, IL-6, and IL-8 were produced at significantly higher levels compared to the control (non-stimulated cells, medium only) when fungal EVs were present in the medium. However, TNF-α levels, while higher than the negative control, were lower compared to the other pro-inflammatory cytokines (Fig. 8A, C, D, and F). When comparing phylogenetically closely related fungal genera, THP-1 cells stimulated with *C. albicans* EVs produced more IL-6 than those stimulated with *C. auris* EVs, whereas *C. auris* EVs induced higher levels of IL-1β and TNF-α. In the *Cryptococcus* group, EVs from *C. neoformans* stimulated higher levels of IL-1β and IL-8 compared to EVs from *C. gatti*. Regarding the anti-inflammatory cytokines, IL-4 levels remained below the control for all EV stimuli, with very low concentrations being detected. Conversely, IL-10 was produced at levels significantly higher than in control cells (Fig. 8B and E). Taken together, these findings suggest a predominance of a pro-inflammatory response mediated by THP-1 cells in the presence of fungal EVs.

## DISCUSSION

Crosstalk can be defined as an exchange of signals between communicating organisms, even over a distance, functioning as a shared “language” based on extracellular signals. These signals are interpreted to coordinate critical processes, such as the survival of pathogenic fungi during infection establishment (37). In fungal communication, essential biological functions are influenced by the exchange of these “messages,” including mating, growth, morphological transitions, and virulence (38). Crosstalk in fungi is partially mediated by EVs, nano-sized structures with bilayer membranes that carry proteins, lipids, glycans, pigments, and nucleic acids (11). Our group recently demonstrated the role of EVs from human pathogenic fungi in intraspecies cellular communication, revealing at the molecular level their impact on gene expression and fungal proliferation control (13). However, this fungal network coordination mechanism does not appear to be limited to the same fungal strain or species. Although still an emerging field, growing evidence supports the existence of EV-mediated interspecies communication, showing significant biological effects, such as biofilm formation and drug-resistance phenotypes, following cross-species interaction (22).

EVs were successfully isolated from cultures of *C. albicans, C. auris, C. neoformans,* and *C. gattii* using the protocol described in Reis et al. (39). The structure and size of fungal EVs were confirmed through NTA, TEM, and cryo-TEM, corroborating findings from several studies (39–42). The combined use of NTA and TEM is of great importance for the characterization of fungal EVs, as these complementary techniques rely on distinct principles: optical and non-optical methods, respectively (43). These studies consistently described fungal EVs as a heterogeneous population of round-shaped structures (44), predominantly around 200 nm in size, a shape that results from collapse during the drying process (45). Although isolation protocols are continuously being improved, some debris resulting from cell lysis, such as membranes and other lipids, may still be present in the samples and consequently detected during TEM visualization (44). Cryo-EM can serve as an alternative to conventional TEM, as the rapid freezing step in sample preparation reduces structural artifacts and data distortion associated with heavy metal staining, dehydration, and chemical fixation (46). Regarding the EV-to-cell ratio, our findings align with observations from other research groups (47), which reported up to 10 EVs per cell in similar isolation protocols.

Zeta potential, a measure of particle surface charge, is a key determinant in the stability and functionality of EVs (48). Under physiological conditions, negatively charged particles, including fungal EVs, exhibit enhanced internalization by phagocytic cells such as macrophages, while positively charged particles tend to be preferentially internalized by non-phagocytic cells (48, 49). In this study, fungal EVs consistently exhibited negative zeta potential values, indicating stable colloidal suspensions with reduced likelihood of aggregation. This characteristic supports their stability in extracellular environments and suggests increased recognition and uptake by innate immune cells. Similar findings in nanoparticle systems have shown that negative zeta potential reduces protein corona formation, prolongs circulation time, and improves delivery efficiency to target tissues (50). The stability and charge properties of EVs may be leveraged for therapeutic delivery, enhancing biodistribution and targeting of infected or inflamed tissues with elevated phagocytic activity (51). This preferential interaction could be further exploited by engineering fungal EVs to carry antifungal compounds or immune modulators, optimizing their delivery to target sites.

It has been demonstrated that vesicular transport in fungi occurs bidirectionally across the fungal cell wall, allowing the export of EVs to the external environment as well as internalization into intracellular compartments after binding to fungal surface receptors (18, 19). Some studies suggest key processes that may be necessary for EV uptake, including the passage of EVs through cell wall pores approximately 5 nm in size (or up to 400 nm under stress conditions), enzymatic modifications of the cell wall (mediated by enzymes carried by EVs), and transit through specific cellular channels (18, 52). Our group previously reported the uptake and association of radiolabeled EVs by cells of different fungal species, such as *C. albicans, Paracoccidioides brasiliensis,* and *A. fumigatus* (13). In the present study, we demonstrate similar events in *C. albicans*, *C. auris*, *C. neoformans*, and *C. gattii* using a fluorescent lipophilic membrane stain. Additionally, SEM revealed an increase in vesicle-like structures on fungal cell surfaces. The internalization of EVs appears to be a conserved mechanism among different fungal species, suggesting the existence of shared pathways mediating EV uptake. This phenomenon points to an evolutionarily conserved strategy for cell-to-cell communication, possibly involving mechanisms analogous to endocytosis or other energy-dependent uptake processes (53). Although the molecular machinery underlying EV uptake in fungi remains incompletely defined, the recurrence of this process across phylogenetically distinct fungal species suggests that it is not incidental, but rather a regulated and functionally relevant mechanism.

After the EV uptake, fungal cells can be triggered to mount specific responses mediated by molecules transported within these vesicles. For instance, polysaccharides like GXM found in *Cryptococcus* EVs can be assimilated by receptor cells and subsequently decorate their surface (54). Since the vesicle cargo includes polysaccharides, proteins, RNA, DNA, and other bioactive molecules, it is expected that recipient cells respond to their release within the intracellular environment. Our results revealed an increase in the mRNA expression of genes associated with virulence following co-incubation with EVs derived from the same or different fungal species. Among these genes, *LAC1* encodes laccase, an enzyme directly involved in melanin production by *Cryptococcus* spp. (55); *URE1* encodes urease, an enzyme responsible for the hydrolysis of urea into ammonia and carbamate (56); and *ERG11* encodes sterol 14-demethylase, a key enzyme in ergosterol biosynthesis (57). The mRNA expression of all these genes was significantly altered after the addition of EVs to fungal cultures.

*CAP59* is a gene essential for capsule formation, as its absence or mutation results in acapsular mutants (58). In our study, an increase in *CAP59* mRNA expression was conserved in cultures of *cap67*Δ mutants exposed to EVs. However, SEM and India Ink staining did not reveal a restoration of the wild-type capsule phenotype. We propose two possible explanations for the reaction observed in the dot blot analysis: (i) GXM polysaccharides from EVs are being recognized as annexed structures on the cell surface and/or (ii) a thin and irregularly spaced GXM layer might be produced by the acapsular mutant through the translation of *CAP59* mRNA (originating from the wild-type strain and free from missense mutations). While the first explanation is based on the hypothesis of the direct transport of GXM molecules via EVs, the second suggests that EVs likely deliver regulatory molecules (e.g., proteins or small RNAs) that modulate gene expression. In fact, both mechanisms may underlie the presence of GXM on the surface of the acapsular mutant and likely operate concurrently, warranting further investigation into their respective roles in GXM transfer to recipient cells. A similar outcome was reported by Bielska et al. (23), who demonstrated that EVs from the wild-type *C. gattii* R265 strain were insufficient to restore capsule formation in an acapsular mutant (R265ΔCap10). Therefore, even though increased *CAP59* mRNA expression was detected by qPCR, this may reflect an upregulated expression of the mutated gene sequence, which would not translate into effective capsule formation.

Beyond planktonic cells, EVs can also influence fungal biofilms. Biofilm formation in fungi is a multi-stage process that includes adhesion, growth, maturation, and dispersion phases (59). Adhesion is a critical initial step, as fungal pathogens rely on this process to colonize host tissues and initiate infection. Adhesins and their corresponding host cell ligands play a pivotal role in biofilm formation (60). Research on the effects of EVs, particularly during the adhesion phase, is still emerging. To the best of our knowledge, our study is among the first to investigate interspecies communication in biofilm formation, and even potential intergeneric interactions, considering the recent taxonomic reclassification of *C. auris* (61). The relocation of *C. auris* to the genus *Candidozyma* (family Metschnikowiaceae) emphasizes how phylogenetically distant it is from *C. albicans*, reinforcing the relevance of our results. While Zarnowski et al. (62) explored intra- and interspecies communication in *Candida* spp. and observed predominant antagonistic interactions between EVs from one species and biofilms from another, our findings suggest a different interaction pattern between EVs and the biofilms of *C. albicans* and *C. auris*. To address this question, we conducted experiments across a broad range of EV concentrations.

Our results indicate that the concentration of EVs is a determining factor for their biological effects, whether enhancing or even inhibiting biofilm adhesion. Notably, EVs derived from *C. neoformans cap67*Δ mutants did not enhance biofilm adhesion when added, and instead, an opposite effect was observed, with a tendency toward reduced adhesion. We hypothesized that this occurs because GXM polysaccharide, which is transported by EVs from the wild-type strains, is essential for increasing adhesion levels. The absence of GXM in *cap67*Δ mutants is a well-known limiting factor for biofilm formation (63). Beyond species identity, the biological impact of EVs among pathogenic fungi appears to be shaped not only by phylogenetic relatedness but also by the specific composition of their cargo, reflecting evolutionary and functional connections between EV-producing and recipient strains. This was evident in the *cap67*Δ mutant (*C. neoformans* var. *neoformans*), which responded similarly to EVs from *C. neoformans* H99 (var. *grubii*), but showed enhanced growth and biofilm adhesion when exposed to EVs from *C. neoformans* B3501 (var. *neoformans*), with detectable effects at lower EV concentrations and reaching higher adhesion levels. These findings suggest that, although EV-mediated communication appears conserved with respect to particle uptake and cargo transfer, the downstream cellular responses may be influenced by a combination of factors such as strain-specific traits and evolutionary proximity. In agreement with previous reports (22), this indicates that EV-mediated communication does not convey a universally consistent signal within a fungal genus.

Dispersion represents the final stage of the biofilm lifecycle, characterized by the detachment and release of cells (primarily in the yeast form) from the biofilm to colonize new surfaces (64). This process is tightly regulated by various molecules and environmental factors, including pH, nutrient availability, and other external cues. Recently, EVs have been suggested as key regulators of biofilm formation and detachment (65). Previous studies using exogenous EV add-back assays in both intra- and interspecies contexts (21, 22, 64) have demonstrated that EV administration can significantly impact dispersion levels. Our findings align with these observations, showing that EVs modulated biofilm dispersion in different fungal species. Interestingly, our results suggest that EVs may carry a conserved set of core effectors capable of triggering similar responses in fungi of other species. The extracellular matrix (ECM), a key component of biofilm architecture, shields fungal cells from toxins (65). EVs mediate ECM remodeling by delivering enzymes, signaling molecules, and virulence factors that degrade, modify, or reorganize the matrix (66). This process reinforces biofilm structural integrity, facilitates nutrient acquisition, and promotes immune evasion, underscoring its role in environmental adaptation and community dispersal (22). In our study, biofilm assays further supported EV-mediated remodeling, as EVs significantly impacted fungal adhesion, dispersal, and antifungal tolerance.

In *Candida* spp.*,* studies have linked the functional cargo transported by EVs to increased protection against antifungal treatment when EVs are added to susceptible strains (21, 22). Specifically, reduced susceptibility to fluconazole and caspofungin in *C. albicans* biofilms supplemented with EVs has been attributed to their positive role in enhancing the ECM architecture (64–66). In our study, we observed differences in antifungal tolerance between EV-treated and untreated (control) planktonic cells and biofilms of *C. albicans* and *C. neoformans cap67*Δ, corroborating previous studies that reported similar roles for fungal EVs in both intra- and interspecies interactions (22, 40). Chan et al. (40) identified modulation of antifungal tolerance, specifically against amphotericin B, only in interactions between fungi and EVs from the same species (*C. auris*). However, our results revealed that this modulation can also occur at the interspecies level in experiments using fluconazole. The upregulation of *ERG11* expression in *C. albicans* exposed to EVs suggests that increased ergosterol synthesis could be a plausible mechanism underlying the enhanced resistance to azoles via EV-mediated communication. However, considering the broad and complex cargo of EVs, additional synergistic contributions to the observed phenotypic changes cannot be excluded, highlighting the need for further investigation into specific effector molecules involved in this process. Data regarding changes in antifungal tolerance following EV exposure in *Cryptococcus* spp. and other fungal pathogens (particularly in the context of cross-species interactions) remain limited to a few studies, reinforcing the relevance of our findings.

While intraspecies communication was not the primary focus here, its potential role was not addressed for *C. neoformans* H99 and *C. gattii* R265 with their own EVs. Future studies may explore EV-mediated intraspecies communication in wild-type *Cryptococcus* strains to enable direct comparison with the results presented in this study. *Candida* spp./*Candidozyma* spp. and *Cryptococcus* spp. are fungi belonging to distinct taxonomic classes, *Ascomycota* and *Basidiomycota*, respectively, which are phylogenetically distant (2). Even though species of *Cryptococcus*, *C. albicans*, and *C. auris* are not expected to share the same ecological niches, they were utilized in this proof-of-concept study to demonstrate the principle of interspecies communication using three medically significant fungal species. Despite their genotypic and phenotypic differences, this study revealed that interspecies communication mediated by EVs can occur between evolutionarily divergent fungi, resulting in biologically relevant effects on recipient cells.

Pathogenic fungal cells communicate with each other (pathogen-to-pathogen), forming a network during infection to coordinate actions through the transport of virulence factors and other molecules. They also communicate with host cells (pathogen-to-host), establishing an inter-kingdom interaction that modulates the host immune response (67). Previous studies, including work from our group, have shown that EVs from *Candidozyma haemuli* (formerly *Candida haemulonii*), *C. albicans,* and *C. neoformans* do not exhibit cytotoxic effects on macrophages. Despite this, immune cells remained capable of responding to EV stimuli through the production of cytokines, antibodies, and antimicrobial compounds, among other defense mechanisms (16, 67). Additionally, some researchers have suggested a correlation between EV size and cytotoxicity, with sEVs being less cytotoxic than lEVs (49). Understanding the internalization of fungal EVs by innate immune cells is a rapidly evolving field. In our study, we contributed to this growing body of knowledge by demonstrating the ability of human macrophages to internalize and elicit specific responses against EVs derived from four different species of pathogenic fungi. Furthermore, our findings indicate low or absent cytotoxicity of the tested fungal EVs. This was supported by the lack of significant statistical differences in macrophage viability and cellular properties when comparing treated and untreated groups.

Pathogens invade the host and release EVs to facilitate communication between pathogenic cells, exchanging “messages” about the local environment. These messages can be intercepted by immune system cells, triggering a response against the intruder (68). The stimulation of macrophages by fungal EVs can affect cellular signaling pathways in diverse ways. This process begins with recognition by pattern recognition receptors and may culminate, for example, in the induction of macrophage polarization into M1 (classically activated) or M2 (alternatively activated) phenotypes (69). M1-polarized macrophages are characterized by the release of high levels of pro-inflammatory cytokines, such as IL-1β, IL-6, TNF-α, and IFNγ, and exhibit potent intracellular pathogen-killing activity mediated by inducible nitric oxide synthase (*iNOS*) and reactive oxygen species (ROS) (70). In contrast, M2 macrophages can be marked by the expression of *ARG1* and produce significant levels of anti-inflammatory cytokines, such as IL-10 and TGF-β. These cells are primarily involved in tissue repair, remodeling, and suppression of the immune response (70, 71). ARG1 competes with nitric oxide synthase for the substrate L-arginine, converting it into urea and ornithine. This effect contrasts with *iNOS*, which converts L-arginine into citrulline and NO (72).

Our results suggest that THP-1 macrophage-like cells were polarized toward the M1 phenotype following stimulation with fungal EVs. We associate macrophage activation with signaling events triggered by EV-transported molecules and with the immune recognition of EV-associated PAMPs. This hypothesis is supported by the detection of STAT1 increase in THP-1-stimulated cells, since STAT1 signaling is linked to M1 polarization with a pro-inflammatory role during immune response (73). These processes lead to biological effects that are evident in both transcriptional activation analyses and cytokine production. This pro-inflammatory behavior has also been reported for EVs from *Candida* spp. (30, 48, 74), *Cryptococcus* spp. (34), and other fungi such as *Aspergillus flavus, A. fumigatus, P. brasiliensis,* and *Trichophyton interdigitale,* among others (14, 15, 17, 75). Oliveira et al. (33) associated stimulation of immune cells with *C. neoformans* EVs with an anti-inflammatory response. However, our findings revealed the opposite effect, a discrepancy that may be attributed to differences in the cytokines evaluated and experimental conditions. As highlighted by Brandt et al. (65), the effects of *C. neoformans* EVs on immune cell interactions remain underexplored, representing a significant research gap that our study aimed to address.

Soluble factors released by fungi into the culture supernatant (i.e., into the extracellular environment), such as proteins, polysaccharides, and metabolic byproducts, can elicit immune responses and may confound the effects attributed exclusively to EVs (76–78). Although the use of EV-depleted supernatants could serve as an appropriate control in assays involving fungus–fungus communication or immune cell stimulation, this condition is not commonly employed. Therefore, future experiments will include EV-depleted supernatants as an additional control, representing a methodological refinement aimed at more precisely delineating the immunological roles of fungal EVs.

While fungal recognition by immune system cells is a well-explored topic, the recognition of EVs remains underexplored. In line with observations by Honorato et al. (79), we also detected enhanced expression of *TLR4* following stimulation with *C. albicans* EVs as well as *C. auris* EVs. This response may be linked to EV surface decorations, including *O-* and *N-*linked mannans, chitin-related structures, and β-1,3-glucans (79). TLR2 and Dectin-1 have been suggested as key receptors involved in fungal EV recognition (17, 80, 81). In our study, we observed species-specific variations in receptor expression: Dectin-1 (*CLEC7A*) was upregulated by *C. albicans* EVs, while *TLR2* was upregulated by *C. auris* and *C. neoformans* EVs. *MYD88* activation is a downstream process event in the TLR2 and TLR4 (67) signaling pathways, aligning with our findings. The detection of distinct receptor activation (or inhibition) profiles may stem from structural differences in EVs or reflect species- and strain-specific strategies employed by pathogenic fungi to evade the immune responses (67, 80). The expression of *TLR9* was a particularly noteworthy finding in our study for two reasons: (i) EVs from *C. albicans* and *C. auris* significantly upregulated *TLR9,* and (ii) *C. gattii* EVs significantly downregulated *TLR9*. Based on Brown et al. (3) and Vargas et al. (82), we hypothesize that the enhanced expression of *TLR9* in response to EVs from *Candida* and *Candidozyma* yeasts may be associated with chitin and nucleic acids present in these vesicles. Conversely, the downregulation of *TLR9* by *C. gatti* EVs may represent an immune evasion strategy, as TLR9-mediated signaling cascades are crucial for host resistance against *C. gatti* infections (83).

Activation of TLRs (TLR2, TLR4, and TLR9) in macrophages leads to downstream MyD88-dependent signaling that can activate STAT1, consistent with its role in TLR signal transduction and inflammatory responses (84). This mechanism may relate to our observations on mRNA expression and protein production after THP-1 stimulation with fungal EVs. TBK1 plays several roles in immunity, and more recently, its function in antifungal immunity has been demonstrated through translocation to phagosome followed by activation of tyrosine kinase SRC, which stimulates the production of pro-inflammatory cytokines and chemokines (85). As observed in our study and recently by Kwaku and colleagues (53), EVs from fungal pathogens can also stimulate an enhanced production of TBK1, an effect that may be associated with the recognition of fungal EVs by TLRs, recruitment by Galectin-3 in phagosomes, STING pathway activation, among others (53, 86).

The immunomodulatory role of Galectin-3 has previously been described in the control of *C. neoformans* and *P. brasiliensis* infections, where its upregulation was observed in infected animals. This lectin was shown to impact EV disruption, inhibit fungal growth, and enhance EV uptake by macrophages (87, 88). Our results revealed an increased expression of *LGALS3* when THP-1 cells were stimulated with fungal EVs, suggesting the involvement of this lectin not only in the recognition and destabilization of *C. neoformans* EVs but also in EVs derived from *C. albicans, C. auris,* and *C. gattii*. The recognition of *Candida* species by immune cells is typically associated with the Dectin-1 receptor and mediation through the Syk/CARD9 signaling pathway, where Syk appears to play a more direct role in antifungal immunity than CARD9 (89). However, it is also possible to observe a CARD9-dependent but Syk-independent response during *C. albicans* recognition. This mechanism involves the intracellular receptor NOD2 (nucleotide-binding oligomerization domain 2) (89, 90), which may partially explain the observed increase in *CLEC7A* and *CARD9* expression alongside reduced *SYK* expression in macrophages stimulated by *C. albicans* EVs.

The contribution of STING (stimulator of interferon genes) during fungal infections remains under discussion, as its activation has been shown to play a negative modulatory role in antifungal immunity following recognition of fungal EVs (91–93). Harding et al. (93) used *C. albicans* EVs to stimulate bone marrow-derived macrophages for 6 h, observing activation of the cGAS (cyclic GMP-AMP synthase)-STING pathway. However, our results demonstrated *STING* mRNA downregulation in macrophages stimulated with all fungal EVs tested. We propose that this discrepancy may stem from several factors, including differences in fungal strains, vesicle cargo composition (which depends on fungal culture conditions), cell lineages, macrophage stimulation duration (6 h vs 20 h), immunomodulatory effects of fungal EVs, or even a macrophage strategy to mitigate excessive STING activation, which could be detrimental to immune cells. In summary, immune responses and activated pathways are both fungal- and EV composition-dependent (94). In addition to STING, cGAS is another key component of this pathway. Its downregulation has been linked to increased levels of pro-inflammatory cytokines (IL-1β, IL-6, and TNF-α) in the context of *A. fumigatus* infections (95). Following THP-1 stimulation with EVs, *C. neoformans* and especially *C. gattii* EVs induced *CGAS* downregulation. This suppression mechanism may negatively impact the immune response, facilitating fungal immune evasion, as suggested by Gu et al. (91) and Peng et al. (95).

The characterization of immune response following exposure of host innate immune cells to fungal EVs has advanced through cytokine secretion analysis, enabling the identification of patterns suggestive of pro- or anti-inflammatory responses (68). EVs from *C. neoformans, C. gattii, C. albicans,* and *C. auris* were previously described as elicitors of pro-inflammatory effects in immune cells (32). These responses typically involved increased production of TNF-α, IL-6, IL-8, and IL-12, alongside reduced or less-pronounced production of TGF-β and IL-10 (67). Consistent with previously published data on fungal EV immunomodulation, our results indicated a predominance of pro-inflammatory cytokines (IL-1β, IL-6, IL-8, and TNF-α) in EV-treated macrophages, accompanied by reduced IL-4 levels. Similarly, decreased anti-inflammatory cytokine production compared to non-treated macrophages has been reported in other studies (48, 74, 96). Despite the overall pro-inflammatory profile, IL-10 secretion was observed at lower levels. A pro-inflammatory response following EV recognition may represent a preparatory mechanism to combat fungal infections (68). This inter-kingdom modulation between host defense mechanisms and pathogenic fungi highlights a crucial area for further exploration, potentially guiding novel interventions for patients affected by invasive fungal infections (97).

Taken together, the results obtained in this study represent valuable tools for the development of antifungal therapies. We demonstrated that fungal cells could communicate with each other via signals carried by EVs, which are interpreted by receptor cells from the same or different species. Although the fungal species studied here are unlikely to be found together in the environment or during infection, they belong to fungal genera that include major human pathogenic fungi and thus serve as representative examples to explore EV-mediated interspecies communication. Using *C. albicans, C. auris, C. neoformans,* and *C. gattii* as pathogenic fungi models, along with their respective EVs, we showed that these molecular messages can also be directed to or intercepted by immune system cells, exerting significant effects on various targets of the innate immune response. Therefore, exploring fungal EVs provides essential insights into fungal communication networks, underscoring the importance of fully elucidating the mechanisms driving fungal coordination during infection.

## MATERIALS AND METHODS

### Strains and culture conditions

*Candida albicans* ATCC 64548 and a clinical isolate of *Candidozyma auris* (synonym *Candida auris*) 470/2015 (clade IV) were cultured in Sabouraud liquid or solid medium (2% wt/vol agar) at 37°C for 48 h, with or without shaking at 200 rpm. *Cryptococcus neoformans* var. *grubii* H99, *Cryptococcus gattii* R265 (kindly supplied by Prof. Dr. Thiago Aparecido da Silva, UNESP), *Cryptococcus neoformans* var. *neoformans* B3501, and the *Cryptococcus neoformans* var. *neoformans* mutant for *CAP67* (*cap67*Δ) were cultured in a chemically defined minimal medium containing 15 mM dextrose, 10 mM MgSO_4_, 29.4 mM KH_2_PO_4_, 13 mM glycine, and 3 μM thiamine. These cultures were incubated under the same conditions described above, as minimal media are associated with increased EV production (98). The *cap67*Δ mutant carries a missense mutation in the *CAP59* gene (position 1345), resulting in an amino acid substitution from glycine (Gly) to serine (Ser). This mutation eliminates capsule formation, resulting in an acapsular strain (Cap^-^ phenotype) (36, 58).

The human monocytic cell line THP-1 (ATCC TIB202) was cultured in RPMI 1640 medium (Gibco) supplemented with 10% (vol/vol) exosome-depleted fetal bovine serum (FBS) (Vitrocell), sodium bicarbonate, and penicillin/streptomycin (1,000 IU). Cells were maintained in a humidified incubator at 37°C with 5% CO_2_. Differentiation of THP-1 monocytes into adherent macrophage-like cells was induced by treating the cultures with 10 ng/mL phorbol 12-myristate 13-acetate (PMA) for 24 h.

### Isolation of EVs

EVs from all yeast strains used in this study were isolated following the protocol described by Reis et al. (39). Briefly, overnight cultures of each yeast strain in 10 mL of Sabouraud medium were counted using a Neubauer chamber. A total of 3,5 × 10^7^ cells were inoculated onto each 2% agar Sabouraud plate, with 10 plates per strain used to ensure sufficient EV yield. After 48 h of incubation at 37°C, yeast material was recovered from the plates using phosphate-buffered saline (PBS). The eluate was centrifuged in a Sorvall LEGEND RT+ (Thermo) centrifuge at 5,000 × *g* for 15 min to remove cells and larger debris, and the pelleted cells were counted to determine the EV-to-cell ratio. The resulting supernatant was further centrifuged at 15,000 × *g* for 15 min to eliminate residual cellular material. The supernatant was passed through 0.45 μm membrane filters (CORNING) to remove any remaining debris. Finally, EVs were pelleted by ultracentrifugation at 100,000 × *g* for 1 h at 4°C using an Optima MAX-XP ultracentrifuge (Beckman). The EV-enriched pellet was resuspended in PBS (Sigma) and stored at −80°C. Subsequent EV analyses were performed in accordance with the MISEV 2023 guidelines (Minimal Information for Studies of Extracellular Vesicles) (12).

### NTA

NTA was conducted to characterize the size distribution and quantify fungal EVs. Analysis was performed on a Nanosight NS300 (Malvern) equipped with NTA 3.0 software, following the manufacturer’s instructions. Samples were diluted at a 1:100 ratio in PBS and carefully loaded into the Nanosight system. System parameters were adjusted to detect 20–100 particles per frame, with camera sensitivity set to 14 or above (16). Results of NTA were plotted using OriginPro 2024 software (OriginLab ).

### Zeta potential

The zeta potential of fungal EVs was measured to evaluate surface charge and colloidal stability, using laser scattering with a Zetasizer Nano ZS (Malvern) (λ = 580 nm, scattering angle 172°), equipped with Zetasizer software v.7.1 (Malvern). Measurements were performed at 25°C in a disposable DTS1070 cuvette using PBS as the dispersant (pH 7.4). Electrophoretic mobility values were converted to zeta potential using the Smoluchowski equation, and each sample was analyzed in triplicate with 10–100 runs per measurement.

### TEM and cryo-EM

TEM was performed with fungal EVs at a concentration of 1 × 10^9^ particles, determined by NTA. Samples were fixed in a solution containing 2% glutaraldehyde and 2% paraformaldehyde in 0.1 M sodium cacodylate buffer (pH 7.4) for 4 h at 4°C. Following fixation, samples were ultracentrifuged at 100,000 × *g* at 4°C for 1 h, and the resulting pellets were resuspended in 200 μL of sodium cacodylate buffer. EV structures were visualized using a JEM 100CXII transmission electron microscope (JEOL), and images were acquired with a Hamamatsu ORCA-HR digital camera (JEOL).

Fungal EVs also had structures (shape and size) characterized by cryo-EM. Suspensions of 1 × 10^12^ fungal EVs (up to 4 μL) were first placed on glow-discharged 300-mesh R1.2/1.3 Quantifoil copper grids (Quantifoil) and vitrification was carried out using a Vitrobot Mark IV (Thermo) operated at 4°C and 100% relative humidity, with blot time and force of 4 s and −2, respectively, followed by rapid plunge-freezing into liquid ethane cooled by liquid nitrogen. The samples containing *Candida, Candidozyma*, and *Cryptococcus* EVs were screened using the Talos Arctica cryo-electron microscope (Thermo) operated at 200 kV, equipped with a Ceta camera (Thermo), at the LNNano facility (CNPEM/MCTI).

### Internalization of EVs by fungal cells and human macrophages

The recognition and internalization of EVs secreted by either the same or different fungal species were analyzed using fluorescence microscopy. A volume corresponding to 1 × 10^9^ EVs was stained with 1 μL of Vybrant DiI Cell-Labeling Solution (5 μM, Invitrogen), a lipophilic fluorescent dye for membrane labeling, and incubated for 20 min at 37°C. After staining, excess dye was removed by washing with PBS. The samples were subsequently ultracentrifuged at 100,000 × *g* at 4°C for 1 h, and the resulting EV pellet was resuspended in Sabouraud liquid medium. Then, EVs were added to fungal cultures at a 1,000:1 EV-to-fungus ratio, which were incubated for EV uptake under agitation at 200 rpm at 37°C.

DiI-stained EVs were used to assess EV internalization by THP-1 cells. THP-1 monocytes (1 × 10⁶ cells/well) were seeded in six-well plates containing RPMI medium supplemented with PMA to induce differentiation into macrophages. After an overnight incubation at 37°C, macrophage-like THP-1 cells were co-incubated with stained EVs previously diluted in RPMI medium to a final concentration of 1 × 10^9^ EVs/mL, and 1 mL of this solution was added to each well, resulting in an EV-to-macrophage ratio of 1,000:1. Following 20 h of incubation at 37°C, fluorescence signals from internalized/associated EVs in fungal cells and macrophages were visualized using an Eclipse Ts2 inverted microscope (Nikon) equipped with a Digital Sight 10 camera and a 560 nm fluorescence filter. Images were analyzed in the NIS-Elements (Nikon) software.

### SEM

SEM was employed to visualize fungal cell morphology following incubation of *C. albicans* and *C. neoformans* (*cap67*Δ mutant) with EVs in Sabouraud medium at a concentration of 1 × 10^9^ EVs and 1 × 10^6^ cells (1,000:1 EV-to-fungus ratio) under the following conditions: (i) *C. albicans* with EVs from *C. auris*, and (ii) *C. neoformans cap67*Δ with EVs from *C. gattii*. Incubation was performed at 37°C with agitation at 200 rpm over 24 h. After incubation, fungal cells were centrifuged at 5,000 × *g* for 15 min and washed with PBS to remove unbound EVs. Cells were then fixed with 2% paraformaldehyde diluted in PBS for 2 h at 4°C, centrifuged again as previously described, and resuspended in a buffer containing 1% osmium tetroxide (OsO_4_). Samples underwent dehydration through a graded ethanol series, followed by critical point drying using an Autosamdri-810 system (Tousimis). Dried samples were mounted on aluminum stubs with silver paint and coated with gold using a Sputter Coater SCD 050 (Bal-Tec). Finally, the prepared samples were visualized using a JSM-6610 LV scanning electron microscope (JEOL).

### Growth curves of pathogenic yeasts co-incubated with EVs

*C. albicans* and *C. auris* were cultured overnight at 37°C in 5 mL of Sabouraud medium, while *C. neoformans cap67*Δ was grown on minimal medium. Cells were counted using a counting chamber and standardized to 1 × 10^6^ cells/mL in fresh Sabouraud or minimal medium, then incubated in microplates in a proportion of 1 × 10^5^ cells/well and 1 × 10^9^ EVs/well, overlaid with sterile mineral oil. Growth curves were followed over 48 h at 37°C, with agitation and optical density readings (OD_600nm_) every 30 min. Growth kinetics assays were performed in triplicate and analyzed using a SpectraMax 190 Microplate Reader (Molecular Devices) with SoftMax Pro software (Molecular Devices). The final OD_600nm_ read was used to compare the biological impact on receptor cells after 48 h of incubation with EVs. For all tests, cells with no EVs added to the medium were used as growth controls, and uninoculated medium was used as a blank (negative control).

### Dot blot

A dot blot assay was performed to detect the polysaccharide component (GXM) of EVs from *C. neoformans* B3501, *C. neoformans* H99, and *C. gattii* R265 using an anti-GXM antibody. First, EVs were isolated as previously described, washed with PBS, and analyzed by NTA. Subsequently, 10 μL of each sample at a concentration of 1 × 10^10^ EVs/mL were spotted onto a polyvinylidene difluoride (PVDF) membrane pre-treated with methanol for 5 min. The membrane was subsequently blocked with 5% (wt/vol) skim milk in PBS for 1 h and washed three times with PBS-T (PBS + 0.05% Tween 20). Next, the membrane was incubated with the monoclonal antibody 18b7, which specifically binds to GXM (generously provided by Prof. Dr. Anamélia Bocca from FIOCRUZ), for 1 h. Following another washing step, an anti-mouse horseradish peroxidase (HRP)-conjugated secondary antibody (Sigma) was applied for 1 h. After five additional washes with PBS-T, the membrane was treated with a detection solution (Vector Laboratories), resulting in a visible signal indicating the presence of GXM in the samples. Aliquots of EVs isolated from *C. neoformans cap67*Δ, 5% bovine serum albumin (BSA), 10% FBS, and PBS were used as negative controls, while cells of B3501, H99, and R265 strains (1 × 10^7^/mL) were used as positive controls.

The transfer of virulence factors via EVs was investigated by evaluating the transmission of GXM, the primary component of the cryptococcal capsule, to the non-capsular mutant strain of *C. neoformans* (*cap67*Δ). A straightforward and effective assay was employed, wherein *C. neoformans cap67*Δ was incubated with EVs from *C. neoformans* and *C. gattii*. In this assay, *C. neoformans cap67*Δ cells (1 × 10^6^/mL) were incubated with EVs (1 × 10^9^/mL) in Sabouraud medium for 24 h at 37°C with 5% CO_2_. After incubation, the cells were harvested by centrifugation at 8,000 × *g* for 15 min, washed with PBS, and 10 μL of 1 × 10^10^ cells/mL were spotted onto a PVDF. Then, the dot blot technique was performed as described previously, using the 18b7 antibody to detect GXM integrated by *cap67*Δ cells exposed to wild-type EVs.

### Co-incubation of *C. albicans* and acapsular *C. neoformans* planktonic cells with EVs

*C. neoformans cap67*Δ was grown to the stationary phase in Sabouraud broth. A volume corresponding to 5 × 10^6^ cells from the previous culture was inoculated into 5 mL of fresh Sabouraud broth and mixed with 1 × 10^9^ EVs (1:200 cell/EV ratio) isolated from *C. neoformans* H99, *C. gattii* R265, or self-derived EVs from *cap67*Δ. Fungal EVs were added at the start of the experiment (0 h) and again after 6 h of incubation. The experiment was conducted over 24 h at 37°C with shaking at 200 rpm. At designated time points, samples were collected, and India ink staining was performed to visualize potential capsule formation on the cellular surface. Cells were then separated from the culture medium by centrifugation at 8,000 × *g* for 15 min at 4°C and subjected to RNA extraction using the Quick-RNA Fungal/Bacterial Miniprep kit (Zymo Research). The isolated RNA was quantified using a NanoDrop One spectrophotometer (Thermo) and subsequently used to evaluate the expression levels of *LAC1*, *URE1,* and *CAP59* by qPCR*,* with results compared to the control culture (*C. neoformans cap67*Δ without EV addition).

The effect of fungal EVs on planktonic cells of *Candida* was investigated using a similar experimental design. Cultures of *C. albicans* were first grown to the stationary phase in Sabouraud broth. The same broth volume, cell/EV ratio, and incubation conditions previously described were applied. In this assay, EVs isolated from *C. albicans* and *C. auris* were analyzed. Additionally, EVs from *C. auris* cultured in the presence of fluconazole were included for comparison. After incubation, the cell pellet was collected by centrifugation, and total RNA was purified and quantified as described before for the *C. neoformans cap67*Δ experiment. The expression level of *ERG11* was then evaluated by qPCR and compared to a control culture (*C. albicans* without EV treatment).

### Spot test

The effect of GXM-transmitting EVs on *C. neoformans cap67*Δ cells was further investigated using a spot assay to assess potential differences in antifungal tolerance. In this assay, 100 μL of EVs (1 × 10^10^ EVs/mL) derived from *C. neoformans* or *C. gattii* were added to cultures of *C. neoformans cap67*Δ (1 × 10^6^ cells/mL) in 10 mL of Sabouraud broth. The cultures were then incubated at 37°C with shaking at 200 rpm for 24 h. Untreated *C. neoformans cap67*Δ cultures served as controls. Following incubation, the cultures were centrifuged at 8,000 × *g* for 15 min*,* washed with PBS, and subjected to 10-fold serial dilutions, starting from 10^7^ cells/mL in PBS. Subsequently, 10 μL of each dilution was plated on Sabouraud agar (control) and Sabouraud agar supplemented with fluconazole (1 μg/mL–6 μg/mL). Plates were incubated at 37°C for 96 h.

To further explore the effect of EVs on antifungal drug tolerance, a similar spot assay was performed using *C. albicans* (fluconazole-susceptible) cultures. These cultures (1 × 10^6^ cells/mL) were mixed with 100 μL of EVs (1 × 10^10^ EVs/mL) derived from a fluconazole-resistant *C. auris* strain and incubated in 10 mL of Sabouraud broth at 37°C with shaking at 200 rpm for 24 h. Control cultures of *C. albicans* and *C. auris* without EV treatment were also included. Following incubation, the cultures were centrifuged, washed, and serially diluted 10-fold. Ten microliters of each dilution was plated on Sabouraud agar and Sabouraud agar supplemented with fluconazole (1,000 μg/mL). Plates were incubated at 37°C for 96 h.

### Biofilm adhesion

The effect of isolated EVs on biofilm adhesion was evaluated following the protocol described by Zarnowski et al. (21), using a 96-well microplate assay. Each well was inoculated with 1 × 10^6^ cells of *C. albicans* or *C. auris* in RPMI medium supplemented with 2% glucose. Different concentrations of EVs (10-fold dilutions ranging from 10^9^ to 10^1^) were added to the culture medium to assess their impact on biofilm formation. After 90 min of incubation at 37°C with 5% CO_2_, the culture medium was carefully removed to eliminate non-adherent cells. The biofilms were then washed with 100 μL of PBS. Biofilm adhesion was quantified using the MTT (dimethyl-2-thiazol tetrazolium bromide) assay. For this, MTT was prepared in PBS at a final concentration of 0.5 mg/mL and protected from light. A volume of 100 μL of this MTT solution was added to each well, and the plate was incubated for 3 h at 37°C. Cellular dehydrogenase activity in living cells can be followed by the reduction of MTT to formazan, a highly colored compound, due to the action of NADH. The formazan crystals were then dissolved in 100 μL of DMSO (dimethyl sulfoxide), followed by an additional 10 min incubation at 37°C with gentle shaking at 50 rpm. Absorbance was measured at 490 nm using a SpectraMax 190 Microplate Reader spectrophotometer (Molecular Devices).

The effect of EVs on biofilm adhesion of *C. neoformans cap67*Δ was also evaluated based on protocols by Martinez et al. (63) and Benaducci (56), with modifications. Briefly, *C. neoformans cap67*Δ was cultured overnight in Sabouraud broth at 37°C with shaking at 200 rpm. Cells were then counted using a Neubauer chamber, centrifuged at 8,000 × *g* for 15 min, and resuspended in minimal medium (20 mg/mL thiamine, 30 mM glucose, 26 mM glycine, 20 mM MgSO_4_·7H_2_O, 58.8 mM KH_2_PO_4_) to a final concentration of 1 × 10^7^ cells/mL. In a 96-well microplate, 1 × 10^6^ cells were added to each well and mixed with EVs at different concentrations (10-fold dilutions ranging from 10^9^ to 10^1^) in a final volume of 200 μL. After the adhesion stage (4 h), the supernatant was carefully aspirated and discarded, and biofilms were washed with PBS to remove non-adherent fungal cells. Biofilm adhesion was subsequently assessed using the MTT assay, following the procedure described above.

### Biofilm dispersion

The impact of EVs on preformed *Candida* and *Candidozyma* biofilms was assessed using a dispersion assay, following adapted protocols from Zarnowski et al. (21) and Zarnowski et al. (22). The assay was performed in 96-well microplates, with each well inoculated with 100 μL of a *C. albicans* or *C. auris* suspension (1 × 10^7^ cells/mL) in RPMI medium supplemented with 2% glucose. After a 90 min incubation at 37°C with 5% CO_2_, the culture medium was aspirated, and the biofilms were washed with 100 μL of PBS. Fresh RPMI medium was then added, and the biofilms were incubated for an additional 24 h under the same conditions. EVs isolated from *C. albicans* and *C. auris* were prepared at concentrations ranging from 1 × 10^9^ to 1 × 10^7^ EVs/mL in RPMI medium, concentrations previously shown to affect the adhesion stage. These EV solutions were added to the preformed biofilms, followed by a 24 h incubation. Afterward, supernatants containing detached cells were carefully transferred to a new microplate, where the quantity of dispersed cells was assessed using an MTT assay with the reagent at double concentration. Biofilm dispersion was analyzed by comparing EV-treated biofilms with untreated control biofilms.

The dispersion stage, characterized by the detachment of microcolonies or planktonic cells from mature *Cryptococcus* biofilms (99), was also evaluated in response to cryptococcal EVs. Following protocols adapted from studies on *Candida* biofilms (21, 22) and *Cryptococcus* biofilms (56, 63), a dispersion assay was performed in 96-well microplates. Each well was inoculated with 1 × 10^6^ cells of *C. neoformans cap67*Δ in minimal medium. After the adhesion and maturation stages (24 h), supernatants were discarded, and biofilms were washed with PBS to remove non-adherent cells. The medium was then carefully replaced with suspensions containing 1 × 10^9^ EVs from *C. neoformans cap67*Δ, *C. neoformans* B3501, *C. neoformans* H99, or *C. gattii* R265, diluted in minimal medium. Following an additional 4 h incubation, biofilm dispersion was quantified using the same MTT assay protocol applied to *Candida/Candidozyma* biofilms.

### Antifungal susceptibility of biofilms

Biofilm susceptibility to antifungal treatment was evaluated using a vesicle add-back assay, following the protocols described by Karkowska-Kuleta et al. (64) and Zarnowski et al. (21). Biofilms of *C. albicans* were established by inoculating 1 × 10^6^ cells per well in a 96-well microplate. After a 90 min incubation at 37°C in a humidified atmosphere with 5% CO_2_ to allow initial adhesion, the culture medium was aspirated, and biofilms were washed with PBS. Subsequently, 100 μL of RPMI medium containing EVs at concentrations of 1 × 10^9^ EVs/mL were added to the wells, followed by a 2 h incubation at 37°C. Fluconazole was then diluted in RPMI to a final concentration of 100 μg/mL and added to the wells, bringing the total volume to 200 μL per well. Control wells included biofilms without EV treatment. The microplates were incubated for 24 h at 37°C with 5% CO_2_ in a humidified environment without agitation. Biofilm susceptibility to fluconazole was assessed using the MTT assay.

The influence of EVs on cell communication and antifungal susceptibility in *C. neoformans cap67*Δ was evaluated using a microplate assay adapted from Martinez et al. (63). *C. neoformans cap67*Δ cells were cultured in minimal medium for 48 h at 37°C with shaking at 200 rpm. The cultures were centrifuged at 8,000 × *g* for 15 min, and the resulting pellet was washed with PBS, centrifuged again, and resuspended in minimal medium to a final concentration of 1 × 10^7^ cells/mL. In a 96-well flat-bottom microplate, each well received 100 μL of a *C. neoformans cap67*Δ suspension (1 × 10^6^ cells/well) in minimal medium and was incubated for 4 h at 37°C. Next, 100 μL of EVs (1 × 10^9^ EV/mL) derived from either *C. neoformans* strains or *C. gattii* were added to the wells*,* followed by a 2 h incubation at 37°C. Antifungal susceptibility changes in *cap67*Δ cells after the co-incubation with EVs were analyzed with the addition of 100 μL of fluconazole at 10 μg/mL. The microplates were incubated at 37°C for 24 h. Absorbance was measured at 600 nm (OD_600nm_) using a SpectraMax 190 Microplate Reader (Molecular Devices).

### Stimulation assay of THP-1 macrophages with fungal EVs

For the stimulation assay, 6-well plates were prepared with 1 × 10^6^ THP-1 cells per well, using RPMI medium supplemented with 10% FBS (EVs-depleted) and PMA, performed in triplicate. Cells were incubated for 24 h at 37°C with 5% CO_2_. EVs from *C. albicans, C. auris, C. neoformans* (H99), and *C. gattii* were diluted in RPMI medium to a final concentration of 1 × 10^9^ EVs/mL, and 1 mL of this solution was added to each well, resulting in an EV-to-macrophage ratio of 1,000:1. Lipopolysaccharide (Sigma) at 100 ng/mL was included as a positive control for immune response activation, while RPMI medium without EVs served as the negative control. The plates were incubated for 20 h at 37°C with 5% CO_2_. To evaluate the impact of EVs on THP-1 cells, cell size, viability, and concentration were assessed using the Countess 3.0 Automated Cell Counter (Thermo). Following incubation, supernatants were carefully collected and stored at −20°C for subsequent cytokine quantification. Cells were harvested using cold PBS and were evaluated for mRNA expression by quantitative real-time PCR or protein production by Western blot. Total RNA was extracted using the RNeasy Mini kit (Qiagen) according to the manufacturer’s protocol. RNA concentration and purity were determined using a Nanodrop One spectrophotometer (Thermo), and the extracted RNA was stored at −80°C for later qPCR analysis.

### Detection of cytokine production

Culture supernatants were pooled according to the respective stimulus and stored at −20°C until cytokine quantification via enzyme-linked immunosorbent assay (ELISA). Human cytokine detection kits for TNF-α, IL-1β, IL-4, IL-6, IL-8, and IL-10 (BD Biosciences) were used following the manufacturer’s instructions. Cytokine levels were determined by measuring absorbance at 450 nm, with wavelength correction at 570 nm, using a SpectraMax 190 Microplate Reader (Molecular Devices).

### Quantitative real-time PCR (qPCR)

For cDNA synthesis, 1 μg of total RNA obtained from the THP-1 stimulation assay was reverse-transcribed using the High-Capacity cDNA Reverse Transcription Kit (Applied Biosystems), following the manufacturer’s protocol, including the recommended time and temperature conditions. Quantitative PCR (qPCR) was performed using the SyGreen Mix (PCR Biosystems) in a final reaction volume of 10 μL per well, containing 50 ng of cDNA. Reactions were conducted on a StepOne Plus Real-Time PCR system (Applied Biosystems) under the following cycling conditions: initial denaturation at 95°C for 2 min, followed by 40 cycles of 5 s at 95°C and 30 s at 60°C, as per PCR Biosystems recommendations. In the fungal communications experiments (fungal culture + EVs), the mRNA expression levels of *ERG11*, *LAC1, URE1,* and *CAP59* were normalized to the housekeeping gene *18S rRNA*. Additionally, the immune response triggered by fungal EVs in THP-1 macrophage cultures was evaluated via qPCR by analyzing the expression of *TLR2, TLR4, TLR9, LGALS3* (Galectin-3)*, CLEC7A* (Dectin-1), *ARG1, iNOS*, *CGAS, STING, SYK, MYD88*, and *CARD9* (refer to Table S1 for primers list). These genes were normalized against the housekeeping gene *ACT1* (β-actin). Relative mRNA expression levels were calculated using the 2^-ΔΔCT^ method, as described previously (100).

### SDS-PAGE and Western blot

Total cell lysates were obtained from fungal EV-stimulated THP-1, suspending cells in lysis buffer (50 mM Tris-HCl, pH 7.5, 150 mM NaCl, 10% vol/vol glycerol, 5 mM EDTA, 1% vol/vol Triton X-100) supplemented with a protease inhibitor cocktail (P8340, Sigma) for 20 min at 4°C. After this incubation, cell lysates were centrifuged at 1,400 × *g* for 20 min at 4°C, and the supernatant was recovered, quantified by Bradford protein assay (Bio-Rad), and stored at −80°C.

SDS-PAGE was conducted with samples equalized to a protein concentration of approximately 50 µg, which were eluted in sample buffer containing 160 mM Tris-HCl (pH 6.8), 4% SDS, 20% glycerol, 100 mM DTT, and 0.005% bromophenol blue. Samples were boiled and applied to a 10% SDS-PAGE gel to separate the protein content based on their molecular weight, then were transferred to a nitrocellulose membrane (Millipore). First, the membranes were incubated with Ponceau S (Merck) to stain separated proteins, photographed, and washed using PBS-T (PBS + 5% Tween 20) to eliminate staining. Next, membranes were blocked for 1 h at room temperature with 5% skim milk diluted in PBS, washed with PBS-T, followed by overnight incubation at 4°C with antibodies for STAT1 (#9172, Cell Signaling) or TBK1 (#3504, Cell Signaling) diluted in PBS + 1% BSA. After sequential washes with PBS-T, a secondary antibody anti-rabbit conjugated with HRP (#31460, Invitrogen) was diluted in PBS + 5% skim milk and used for 1 h in an incubation at room temperature. The membranes were washed again, then protein production was detected using a combination of chemiluminescence solutions formulated with (i) 1M Tris-HCl (pH 8.5), 250 mM luminol, 90 mM p-coumaric acid, and (ii) 30% H_2_O_2_, 1 M Tris-HCl (pH 8.5), with a posterior visualization using a ChemiDoc imaging system (Bio-Rad).

### Statistical analysis

Data analysis was performed using GraphPad Prism 9.0 (Dotmatics). Statistical comparisons were conducted using either one-way or two-way ANOVA, depending on the experimental design. A *P*-value of *P* < 0.05 was considered statistically significant.

## SUPPLEMENTAL MATERIAL

Supplemental material mbio.03469-25-s0001.docx Supplemental figures and table.

ASM does not own the copyrights to Supplemental Material that may be linked to, or accessed through, an article. The authors have granted ASM a non-exclusive, world-wide license to publish the Supplemental Material files. Please contact the corresponding author directly for reuse.

## References

[1] Jafarlou M. 2024. Unveiling the menace: a thorough review of potential pandemic fungal disease. Front Fungal Biol 5:1338726. doi:10.3389/ffunb.2024.1338726 38711422 PMC11071163

[2] Rokas A. 2022. Evolution of the human pathogenic lifestyle in fungi. Nat Microbiol 7:607–619. doi:10.1038/s41564-022-01112-0 35508719 PMC9097544

[3] Brown GD, Ballou ER, Bates S, Bignell EM, Borman AM, Brand AC, Brown AJP, Coelho C, Cook PC, Farrer RA, Govender NP, Gow NAR, Hope W, Hoving JC, Dangarembizi R, Harrison TS, Johnson EM, Mukaremera L, Ramsdale M, Thornton CR, Usher J, Warris A, Wilson D. 2024. The pathobiology of human fungal infections. Nat Rev Microbiol 22:687–704. doi:10.1038/s41579-024-01062-w 38918447

[4] Seidel D, Wurster S, Jenks JD, Sati H, Gangneux JP, Egger M, Alastruey-Izquierdo A, Ford NP, Chowdhary A, Sprute R, Cornely O, Thompson GR, Hoenigl M, Kontoyiannis DP. 2024. Impact of climate change and natural disasters on fungal infections. Lancet Microbe 5:e594–e605. doi:10.1016/S2666-5247(24)00039-9 38518791

[5] Denning DW. 2024. Global incidence and mortality of severe fungal disease. Lancet Infect Dis 24:e428–e438. doi:10.1016/S1473-3099(23)00692-8 38224705

[6] Inácio MM, Moreira ALE, Cruz-Leite VRM, Mattos K, Silva LOS, Venturini J, Ruiz OH, Ribeiro-Dias F, Weber SS, Soares CM de A, Borges CL. 2023. Fungal vaccine development: state of the art and perspectives using immunoinformatics. JoF 9:633. doi:10.3390/jof9060633 37367569 PMC10301004

[7] Fisher MC, Denning DW. 2023. The WHO fungal priority pathogens list as a game-changer. Nat Rev Microbiol 21:211–212. doi:10.1038/s41579-023-00861-x 36747091 PMC9901396

[8] Lionakis MS, Drummond RA, Hohl TM. 2023. Immune responses to human fungal pathogens and therapeutic prospects. Nat Rev Immunol 23:433–452. doi:10.1038/s41577-022-00826-w 36600071 PMC9812358

[9] Chang CC, Harrison TS, Bicanic TA, Chayakulkeeree M, Sorrell TC, Warris A, Hagen F, Spec A, Oladele R, Govender NP, et al.. 2024. Global guideline for the diagnosis and management of cryptococcosis: an initiative of the ECMM and ISHAM in cooperation with the ASM. Lancet Infect Dis 24:e495–e512. doi:10.1016/S1473-3099(23)00731-4 38346436 PMC11526416

[10] Rizzo J, Rodrigues ML, Janbon G. 2020. Extracellular vesicles in fungi: past, present, and future perspectives. Front Cell Infect Microbiol 10:346. doi:10.3389/fcimb.2020.00346 32760680 PMC7373726

[11] de Oliveira HC, Castelli RF, Reis FCG, Rizzo J, Rodrigues ML. 2020. Pathogenic delivery: the biological roles of cryptococcal extracellular vesicles. Pathogens 9:1–14. doi:10.3390/pathogens9090754 PMC7557404 32948010

[12] Welsh JA, Goberdhan DCI, O’Driscoll L, Buzas EI, Blenkiron C, Bussolati B, Cai H, Di Vizio D, Driedonks TAP, Erdbrügger U, et al.. 2024. Minimal information for studies of extracellular vesicles (MISEV2023): From basic to advanced approaches. J of Extracellular Vesicle 13:e12404. doi:10.1002/jev2.12404 PMC10850029 38326288

[13] Bitencourt TA, Hatanaka O, Pessoni AM, Freitas MS, Trentin G, Santos P, Rossi A, Martinez-Rossi NM, Alves LL, Casadevall A, Rodrigues ML, Almeida F. 2022. Fungal extracellular vesicles are involved in intraspecies intracellular communication. mBio 13:e0327221. doi:10.1128/mbio.03272-21 35012355 PMC8749427

[14] Brauer VS, Pessoni AM, Bitencourt TA, de Paula RG, de Oliveira Rocha L, Goldman GH, Almeida F. 2020. Extracellular Vesicles from Aspergillus flavus induce M1 polarization in vitro. mSphere 5:e00190-20. doi:10.1128/mSphere.00190-20 32376699 PMC7203453

[15] Freitas MS, Bitencourt TA, Rezende CP, Martins NS, Dourado T de MH, Tirapelli CR, Almeida F. 2023. Aspergillus fumigatus extracellular vesicles display increased Galleria mellonella survival but partial pro-inflammatory response by macrophages. JoF 9:541. doi:10.3390/jof9050541 37233252 PMC10218922

[16] Oliveira BTM, Dourado TMH, Santos PWS, Bitencourt TA, Tirapelli CR, Colombo AL, Almeida F. 2023. Extracellular vesicles from Candida haemulonii var. vulnera modulate macrophage oxidative burst. JoF 9:562. doi:10.3390/jof9050562 37233272 PMC10219333

[17] Bitencourt TA, Rezende CP, Quaresemin NR, Moreno P, Hatanaka O, Rossi A, Martinez-Rossi NM, Almeida F. 2018. Extracellular vesicles from the dermatophyte Trichophyton interdigitale modulate macrophage and keratinocyte functions. Front Immunol 9:2343. doi:10.3389/fimmu.2018.02343 30356863 PMC6190888

[18] U. Stotz H, Brotherton D, Inal J. 2022. Communication is key: extracellular vesicles as mediators of infection and defence during host–microbe interactions in animals and plants. FEMS Microbiol Rev 46:1–18. doi:10.1093/femsre/fuab044 PMC8767456 34448857

[19] Rodrigues ML, Casadevall A. 2018. A two-way road: novel roles for fungal extracellular vesicles. Mol Microbiol 110:11–15. doi:10.1111/mmi.14095 30079549

[20] Honorato L, de Araujo JFD, Ellis CC, Piffer AC, Pereira Y, Frases S, de Sousa Araújo GR, Pontes B, Mendes MT, Pereira MD, Guimarães AJ, da Silva NM, Vargas G, Joffe L, Del Poeta M, Nosanchuk JD, Zamith-Miranda D, dos Reis FCG, de Oliveira HC, Rodrigues ML, de Toledo Martins S, Alves LR, Almeida IC, Nimrichter L. 2022. Extracellular vesicles regulate biofilm formation and yeast-to-hypha differentiation in Candida albicans. mBio 13:e0030122. doi:10.1128/mbio.00301-22 35420476 PMC9239257

[21] Zarnowski R, Noll A, Chevrette MG, Sanchez H, Jones R, Anhalt H, Fossen J, Jaromin A, Currie C, Nett JE, Mitchell A, Andes DR. 2021. Coordination of fungal biofilm development by extracellular vesicle cargo. Nat Commun 12:6235. doi:10.1038/s41467-021-26525-z 34716343 PMC8556236

[22] Zarnowski R, Sanchez H, Jaromin A, Zarnowska UJ, Nett JE, Mitchell AP, Andes D. 2022. A common vesicle proteome drives fungal biofilm development. Proc Natl Acad Sci USA 119:e2211424119. doi:10.1073/pnas.2211424119 36095193 PMC9501958

[23] Bielska E, Sisquella MA, Aldeieg M, Birch C, O’Donoghue EJ, May RC. 2018. Pathogen-derived extracellular vesicles mediate virulence in the fatal human pathogen Cryptococcus gattii. Nat Commun 9:1556. doi:10.1038/s41467-018-03991-6 29674675 PMC5908794

[24] Rodrigues ML, Nakayasu ES, Oliveira DL, Nimrichter L, Nosanchuk JD, Almeida IC, Casadevall A. 2008. Extracellular vesicles produced by Cryptococcus neoformans contain protein components associated with virulence. Eukaryot Cell 7:58–67. doi:10.1128/EC.00370-07 18039940 PMC2224146

[25] Rodrigues ML, Nimrichter L, Oliveira DL, Nosanchuk JD, Casadevall A. 2008. Vesicular trans-cell wall transport in fungi: a mechanism for the delivery of virulence-associated macromolecules? Lipid Insights 2:27–40. doi:10.4137/lpi.s1000 20617119 PMC2898286

[26] Kassi FK, Drakulovski P, Bellet V, Roger F, Chabrol A, Krasteva D, Doumbia A, Landman R, Kakou A, Reynes J, Delaporte E, Menan HEI, Bertout S. 2019. Cryptococcus genetic diversity and mixed infections in Ivorian HIV patients: a follow up study. PLoS Negl Trop Dis 13:e0007812. doi:10.1371/journal.pntd.0007812 31738768 PMC6886875

[27] de Faria Ferreira M, Brito-Santos F, Henrique Nascimento Theodoro P, de Abreu Almeida M, Lazera M dos S, Trilles L. 2022. Mixed infection by Cryptococcus neoformans and Cryptococcus gattii and coinfection with paracoccidioidomycosis in PLHIV. Med Mycol Case Rep 35:48–50. doi:10.1016/j.mmcr.2022.01.006 35256962 PMC8897171

[28] Ullah A, Huang Y, Zhao K, Hua Y, Ullah S, Rahman MU, Wang J, Wang Q, Hu X, Zheng L. 2023. Characteristics and potential clinical applications of the extracellular vesicles of human pathogenic fungi. BMC Microbiol 23:227. doi:10.1186/s12866-023-02945-3 37598156 PMC10439556

[29] Freitas MS, Bonato VLD, Pessoni AM, Rodrigues ML, Casadevall A, Almeida F. 2019. Fungal extracellular vesicles as potential targets for immune interventions. mSphere 4:1–9. doi:10.1128/mSphere.00747-19 PMC6835212 31694899

[30] Vargas G, Rocha JDB, Oliveira DL, Albuquerque PC, Frases S, Santos SS, Nosanchuk JD, Gomes AMO, Medeiros L, Miranda K, Sobreira TJP, Nakayasu ES, Arigi EA, Casadevall A, Guimaraes AJ, Rodrigues ML, Freire-de-Lima CG, Almeida IC, Nimrichter L. 2015. Compositional and immunobiological analyses of extracellular vesicles released by Candida albicans. Cell Microbiol 17:389–407. doi:10.1111/cmi.12374 25287304

[31] de Rezende CP, Santos PWS, Piraine RA, Silvestrini VC, Barbosa JCJ, Valera FCP, Tamashiro E, Podolski‐Gondim GG, Quintana SM, Calado R, Martinez R, Fill TP, Rodrigues ML, Almeida F. 2025. Extracellular vesicles from fungal infection in humans: a key player in immunological responses. J of Extracellular Bio 4:e70065. doi:10.1002/jex2.70065 40881815 PMC12381960

[32] Lai Y, Jiang B, Hou F, Huang X, Ling B, Lu H, Zhong T, Huang J. 2023. The emerging role of extracellular vesicles in fungi: a double-edged sword. Front Microbiol 14:1–15. doi:10.3389/fmicb.2023.1216895 PMC10390730 37533824

[33] Oliveira DL, Freire-de-Lima CG, Nosanchuk JD, Casadevall A, Rodrigues ML, Nimrichter L. 2010. Extracellular vesicles from Cryptococcus neoformans modulate macrophage functions. Infect Immun 78:1601–1609. doi:10.1128/IAI.01171-09 20145096 PMC2849392

[34] Marina CL, Bürgel PH, Agostinho DP, Zamith-Miranda D, Las-Casas L de O, Tavares AH, Nosanchuk JD, Bocca AL. 2020. Nutritional conditions modulate C. neoformans extracellular vesicles’ capacity to elicit host immune response. Microorganisms 8:1815. doi:10.3390/microorganisms8111815 33217920 PMC7698703

[35] De Jesus M, Chow S-K, Cordero RJB, Frases S, Casadevall A. 2010. Galactoxylomannans from Cryptococcus neoformans varieties neoformans and grubii are structurally and antigenically variable. Eukaryot Cell 9:1018–1028. doi:10.1128/EC.00268-09 20061411 PMC2901672

[36] Chang YC, Kwon-Chung KJ. 1994. Complementation of a capsule-deficient mutation of Cryptococcus neoformans restores its virulence. Mol Cell Biol 14:4912–4919. doi:10.1128/mcb.14.7.4912-4919.1994 8007987 PMC358863

[37] Zapparata A, Baroncelli R, Brandström Durling M, Kubicek CP, Karlsson M, Vannacci G, Sarrocco S. 2021. Fungal cross-talk: an integrated approach to study distance communication. Fungal Genet Biol 148:103518. doi:10.1016/j.fgb.2021.103518 33497840

[38] Cottier F, Mühlschlegel FA. 2012. Communication in fungi. Int J Microbiol 2012:351832. doi:10.1155/2012/351832 21961006 PMC3180779

[39] Reis FCG, Borges BS, Jozefowicz LJ, Sena BAG, Garcia AWA, Medeiros LC, Martins ST, Honorato L, Schrank A, Vainstein MH, Kmetzsch L, Nimrichter L, Alves LR, Staats CC, Rodrigues ML. 2019. A novel protocol for the isolation of fungal extracellular vesicles reveals the participation of a putative scramblase in polysaccharide export and capsule construction in Cryptococcus gattii mSphere 4:e00080. doi:10.1128/mSphere.00080-19 30894430 PMC6429041

[40] Chan W, Chow FWN, Tsang CC, Liu X, Yao W, Chan TTY, Siu GKH, Ho AYM, Luk KS, Lau SKP, Woo PCY. 2022. Induction of amphotericin B resistance in susceptible Candida auris by extracellular vesicles. Emerg Microbes Infect 11:1900–1909. doi:10.1080/22221751.2022.2098058 35786393 PMC9341352

[41] Rodrigues ML, May RC, Janbon G. 2024. The multiple frontiers in the study of extracellular vesicles produced by fungi. Microbes Infect 26:105233. doi:10.1016/j.micinf.2023.105233 37805124

[42] Reis FCG, Gimenez B, Jozefowicz LJ, Castelli RF, Martins ST, Alves LR, de Oliveira HC, Rodrigues ML. 2021. Analysis of cryptococcal extracellular vesicles: experimental approaches for studying their diversity among multiple isolates, kinetics of production, methods of separation, and detection in cultures of titan cells. Microbiol Spectr 9:e0012521. doi:10.1128/spectrum.00125-21 34346749 PMC8552642

[43] van der Pol E, Hoekstra AG, Sturk A, Otto C, van Leeuwen TG, Nieuwland R. 2010. Optical and non-optical methods for detection and characterization of microparticles and exosomes. J Thromb Haemost 8:2596–2607. doi:10.1111/j.1538-7836.2010.04074.x 20880256

[44] Théry C, Amigorena S, Raposo G, Clayton A. 2006. Isolation and characterization of exosomes from cell culture supernatants and biological fluids. Curr Protoc Cell Biol 30:1–29. doi:10.1002/0471143030.cb0322s30 18228490

[45] Raposo G, Stoorvogel W. 2013. Extracellular vesicles: exosomes, microvesicles, and friends. J Cell Biol 200:373–383. doi:10.1083/jcb.201211138 23420871 PMC3575529

[46] Rizzo J, Wong SSW, Gazi AD, Moyrand F, Chaze T, Commere P-H, Novault S, Matondo M, Péhau-Arnaudet G, Reis FCG, Vos M, Alves LR, May RC, Nimrichter L, Rodrigues ML, Aimanianda V, Janbon G. 2021. Cryptococcus extracellular vesicles properties and their use as vaccine platforms. J Extracell Vesicles 10:e12129. doi:10.1002/jev2.12129 34377375 PMC8329992

[47] Rodrigues ML, Nimrichter L. 2022. From fundamental biology to the search for innovation: the story of fungal extracellular vesicles. Eur J Cell Biol 101:151205. doi:10.1016/j.ejcb.2022.151205 35176565

[48] Kulig K, Bednaruk K, Rudolphi-Szydło E, Barbasz A, Wronowska E, Barczyk-Woznicka O, Karnas E, Pyza E, Zuba-Surma E, Rapala-Kozik M, Karkowska-Kuleta J. 2023. Stress conditions affect the immunomodulatory potential of Candida albicans extracellular vesicles and their impact on cytokine release by THP-1 human macrophages. Int J Mol Sci 24:17179. doi:10.3390/ijms242417179 38139005 PMC10742962

[49] Fröhlich E. 2012. The role of surface charge in cellular uptake and cytotoxicity of medical nanoparticles. Int J Nanomedicine 7:5577–5591. doi:10.2147/IJN.S36111 23144561 PMC3493258

[50] Dietz L, Oberländer J, Mateos-Maroto A, Schunke J, Fichter M, Krämer-Albers EM, Landfester K, Mailänder V. 2023. Uptake of extracellular vesicles into immune cells is enhanced by the protein corona. J Extracell Vesicles 12:e12399. doi:10.1002/jev2.12399 38124271 PMC10733601

[51] Liam-Or R, Faruqu FN, Walters A, Han S, Xu L, Wang JTW, Oberlaender J, Sanchez-Fueyo A, Lombardi G, Dazzi F, Mailaender V, Al-Jamal KT. 2024. Cellular uptake and in vivo distribution of mesenchymal-stem-cell-derived extracellular vesicles are protein corona dependent. Nat Nanotechnol 19:846–855. doi:10.1038/s41565-023-01585-y 38366223 PMC11186763

[52] Walker L, Sood P, Lenardon MD, Milne G, Olson J, Jensen G, Wolf J, Casadevall A, Adler-Moore J, Gow NAR. 2018. The viscoelastic properties of the fungal cell wall allow traffic of ambisome as intact liposome vesicles. mBio 9:e02383-17. doi:10.1128/mBio.02383-17 29437927 PMC5801470

[53] Kwaku GN, Jensen KN, Simaku P, Floyd DJ, Saelens JW, Reardon CM, Ward RA, Basham KJ, Hepworth OW, Vyas TD, Zamith-Miranda D, Nosanchuk JD, Vyas JM, Brown Harding H. 2025. Extracellular vesicles from diverse fungal pathogens induce species-specific and endocytosis-dependent immunomodulation. PLoS Pathog 21:e1012879. doi:10.1371/journal.ppat.1012879 40445992 PMC12157816

[54] Rodrigues ML, Nimrichter L, Oliveira DL, Frases S, Miranda K, Zaragoza O, Alvarez M, Nakouzi A, Feldmesser M, Casadevall A. 2007. Vesicular polysaccharide export in Cryptococcus neoformans is a eukaryotic solution to the problem of fungal trans-cell wall transport. Eukaryot Cell 6:48–59. doi:10.1128/EC.00318-06 17114598 PMC1800364

[55] Salas SD, Bennett JE, Kwon-Chung KJ, Perfect JR, Williamson PR. 1996. Effect of the laccase gene CNLAC1, on virulence of Cryptococcus neoformans. J Exp Med 184:377–386. doi:10.1084/jem.184.2.377 8760791 PMC2192698

[56] Benaducci T, Sardi J de CO, Lourencetti NMS, Scorzoni L, Gullo FP, Rossi SA, Derissi JB, de Azevedo Prata MC, Fusco-Almeida AM, Mendes-Giannini MJS. 2016. Virulence of Cryptococcus sp. biofilms in vitro and in vivo using Galleria mellonella as an alternative model. Front Microbiol 7:290. doi:10.3389/fmicb.2016.00290 27014214 PMC4783715

[57] Rybak JM, Cuomo CA, Rogers PD. 2022. The molecular and genetic basis of antifungal resistance in the emerging fungal pathogen Candida auris. Curr Opin Microbiol 70:102208. doi:10.1016/j.mib.2022.102208 36242897 PMC10364995

[58] García-Rivera J, Chang YC, Kwon-Chung KJ, Casadevall A. 2004. Cryptococcus neoformans CAP59 (or Cap59p) is involved in the extracellular trafficking of capsular glucuronoxylomannan. Eukaryot Cell 3:385–392. doi:10.1128/EC.3.2.385-392.2004 15075268 PMC387637

[59] Wang D, Zeng N, Li C, Li Z, Zhang N, Li B. 2024. Fungal biofilm formation and its regulatory mechanism. Heliyon 10:e32766. doi:10.1016/j.heliyon.2024.e32766 38988529 PMC11233959

[60] Tronchin G, Pihet M, Lopes-Bezerra LM, Bouchara J-P. 2008. Adherence mechanisms in human pathogenic fungi. Med Mycol 46:749–772. doi:10.1080/13693780802206435 18651303

[61] Liu F, Hu Z-D, Zhao X-M, Zhao W-N, Feng Z-X, Yurkov A, Alwasel S, Boekhout T, Bensch K, Hui F-L, Bai F-Y, Wang Q-M. 2024. Phylogenomic analysis of the Candida auris-Candida haemuli clade and related taxa in the Metschnikowiaceae, and proposal of thirteen new genera, fifty-five new combinations and nine new species. Persoonia 52:22–43. doi:10.3767/persoonia.2024.52.02 39161632 PMC11319837

[62] Zarnowski R, Massey J, Mitchell AP, Andes D. 2022. Extracellular vesicles contribute to mixed-fungal species competition during biofilm initiation. mBio 13:e0298822. doi:10.1128/mbio.02988-22 36377868 PMC9765065

[63] Martinez LR, Casadevall A. 2006. Susceptibility of Cryptococcus neoformans biofilms to antifungal agents in vitro. Antimicrob Agents Chemother 50:1021–1033. doi:10.1128/AAC.50.3.1021-1033.2006 16495265 PMC1426450

[64] Karkowska-Kuleta J, Kulig K, Bras G, Stelmaszczyk K, Surowiec M, Kozik A, Karnas E, Barczyk-Woznicka O, Zuba-Surma E, Pyza E, Rapala-Kozik M. 2023. Candida albicans biofilm-derived extracellular vesicles are involved in the tolerance to caspofungin, biofilm detachment, and fungal proteolytic activity. JoF 9:1078. doi:10.3390/jof9111078 37998883 PMC10672323

[65] Brandt P, Singha R, Ene IV. 2024. Hidden allies: how extracellular vesicles drive biofilm formation, stress adaptation, and host-immune interactions in human fungal pathogens. mBio 15:e0304523. doi:10.1128/mbio.03045-23 39555918 PMC11633191

[66] Zarnowski R, Sanchez H, Covelli AS, Dominguez E, Jaromin A, Bernhardt J, Mitchell KF, Heiss C, Azadi P, Mitchell A, Andes DR. 2018. Candida albicans biofilm-induced vesicles confer drug resistance through matrix biogenesis. PLoS Biol 16:e2006872. doi:10.1371/journal.pbio.2006872 30296253 PMC6209495

[67] Rodrigues ML, Janbon G. 2021. Fungal extracellular vesicles biological roles. Springer International Publishing, Cham.

[68] Kwaku GN, Ward RA, Vyas JM, Harding HB. 2024. Host innate immune systems gather intel on invading microbes via pathogen-derived extracellular vesicles. Extracell Vesicle 3:100043. doi:10.1016/j.vesic.2024.100043 38939756 PMC11209872

[69] Oliveira-Brito PKM, Rezende CP, Almeida F, Roque-Barreira MC, da Silva TA. 2020. iNOS/Arginase-1 expression in the pulmonary tissue over time during Cryptococcus gattii infection. Innate Immun 26:117–129. doi:10.1177/1753425919869436 31446837 PMC7016403

[70] Strizova Z, Benesova I, Bartolini R, Novysedlak R, Cecrdlova E, Foley LK, Striz I. 2023. M1/M2 macrophages and their overlaps – myth or reality? Clin Sci 137:1067–1093. doi:10.1042/CS20220531 PMC10407193 37530555

[71] Wang L-X, Zhang S-X, Wu H-J, Rong X-L, Guo J. 2019. M2b macrophage polarization and its roles in diseases. J Leukoc Biol 106:345–358. doi:10.1002/JLB.3RU1018-378RR 30576000 PMC7379745

[72] Arranz A, Doxaki C, Vergadi E, Martinez de la Torre Y, Vaporidi K, Lagoudaki ED, Ieronymaki E, Androulidaki A, Venihaki M, Margioris AN, Stathopoulos EN, Tsichlis PN, Tsatsanis C. 2012. Akt1 and Akt2 protein kinases differentially contribute to macrophage polarization. Proc Natl Acad Sci USA 109:9517–9522. doi:10.1073/pnas.1119038109 22647600 PMC3386059

[73] Chi Y, Jiang H, Yin Y, Zhou X, Shao Y, Li Y, Rao J. 2025. Macrophage signaling pathways in health and disease: from bench to bedside applications. MedComm 6:e70256. doi:10.1002/mco2.70256 40529613 PMC12171086

[74] Zamith-Miranda D, Heyman HM, Couvillion SP, Cordero RJB, Rodrigues ML, Nimrichter L, Casadevall A, Amatuzzi RF, Alves LR, Nakayasu ES, Nosanchuk JD. 2021. Comparative molecular and immunoregulatory analysis of extracellular vesicles from Candida albicans and Candida auris. mSystems 6:e0082221. doi:10.1128/mSystems.00822-21 34427507 PMC8407381

[75] da Silva TA, Roque-Barreira MC, Casadevall A, Almeida F. 2016. Extracellular vesicles from Paracoccidioides brasiliensis induced M1 polarization in vitro. Sci Rep 6:35867. doi:10.1038/srep35867 27775058 PMC5075875

[76] Bürgel PH, Marina CL, Saavedra PHV, Albuquerque P, de Oliveira SAM, Veloso Janior PH de H, Castro RA de, Heyman HM, Coelho C, Cordero RJB, Casadevall A, Nosanchuk JD, Nakayasu ES, May RC, Tavares AH, Bocca AL. 2020. Cryptococcus neoformans secretes small molecules that inhibit IL-1β inflammasome-dependent secretion. Mediators Inflamm 2020:1–20. doi:10.1155/2020/3412763 PMC7748918 33380899

[77] Wang W, Deng Z, Wu H, Zhao Q, Li T, Zhu W, Wang X, Tang L, Wang C, Cui SZ, Xiao H, Chen J. 2019. A small secreted protein triggers a TLR2/4-dependent inflammatory response during invasive Candida albicans infection. Nat Commun 10:1–14. doi:10.1038/s41467-019-08950-3 30833559 PMC6399272

[78] Huang X-Z, Liang P-P, Ma H, Yi J-L, Yin S-C, Chen Z-R, Li M-R, Lai W, Chen J. 2015. Effect of culture supernatant derived from Trichophyton rubrum grown in the nail medium on the innate immunity-related molecules of HaCaT. Chin Med J 128:3094–3100. doi:10.4103/0366-6999.169106 26608992 PMC4795267

[79] Honorato L, Bonilla JJA, Valdez AF, Frases S, Araújo GR de S, Sabino ALR do N, da Silva NM, Ribeiro L, Ferreira M da S, Kornetz J, Rodrigues ML, Cunningham I, Gow NAR, Gacser A, Guimarães AJ, Dutra FF, Nimrichter L. 2024. Toll-like receptor 4 (TLR4) is the major pattern recognition receptor triggering the protective effect of a Candida albicans extracellular vesicle-based vaccine prototype in murine systemic candidiasis. mSphere 9:e0046724. doi:10.1128/msphere.00467-24 39037263 PMC11351041

[80] Montanari Borges B, Gama de Santana M, Willian Preite N, de Lima Kaminski V, Trentin G, Almeida F, Vieira Loures F. 2024. Extracellular vesicles from virulent P. brasiliensis induce TLR4 and dectin-1 expression in innate cells and promote enhanced Th1/Th17 response. Virulence 15:2329573. doi:10.1080/21505594.2024.2329573 38511558 PMC10962619

[81] Pruksaphon K, Amsri A, Thammasit P, Nosanchuk JD, Youngchim S. 2023. Extracellular vesicles derived from Talaromyces marneffei contain immunogenic compounds and modulate THP-1 macrophage responses. Front Immunol 14:1192326. doi:10.3389/fimmu.2023.1192326 37457708 PMC10339390

[82] Vargas G, Honorato L, Guimarães AJ, Rodrigues ML, Reis FCG, Vale AM, Ray A, Nosanchuk JD, Nimrichter L. 2020. Protective effect of fungal extracellular vesicles against murine candidiasis. Cell Microbiol 22:1–15. doi:10.1111/cmi.13238 PMC7499402 32558196

[83] da Silva-Junior EB, Firmino-Cruz L, Guimarães-de-Oliveira JC, De-Medeiros JVR, de Oliveira Nascimento D, Freire-de-Lima M, de Brito-Gitirana L, Morrot A, Previato JO, Mendonça-Previato L, Decote-Ricardo D, de Matos Guedes HL, Freire-de-Lima CG. 2021. The role of toll-like receptor 9 in a murine model of Cryptococcus gattii infection. Sci Rep 11:1407. doi:10.1038/s41598-021-80959-5 33446850 PMC7809259

[84] Luu K, Greenhill CJ, Majoros A, Decker T, Jenkins BJ, Mansell A. 2014. STAT1 plays a role in TLR signal transduction and inflammatory responses. Immunol Cell Biol 92:761–769. doi:10.1038/icb.2014.51 25027037

[85] Feng Y, Cheng X, Yang Y, Liu C, Shi Y, Qi X, Zhao W, Liu B, Chen T, Gao C. 2025. TBK1 phagosomal recruitment enhances antifungal immunity via positive feedback regulation with SRC. Cell Rep 44:115972. doi:10.1016/j.celrep.2025.115972 40638388

[86] Yu T, Yi Y-S, Yang Y, Oh J, Jeong D, Cho JY. 2012. The pivotal role of TBK1 in inflammatory responses mediated by macrophages. Mediators Inflamm 2012:1–8. doi:10.1155/2012/979105 PMC3523167 23304064

[87] Almeida F, Wolf JM, da Silva TA, DeLeon-Rodriguez CM, Rezende CP, Pessoni AM, Fernandes FF, Silva-Rocha R, Martinez R, Rodrigues ML, Roque-Barreira MC, Casadevall A. 2017. Galectin-3 impacts Cryptococcus neoformans infection through direct antifungal effects. Nat Commun 8:1968. doi:10.1038/s41467-017-02126-7 29213074 PMC5719036

[88] Hatanaka O, Rezende CP, Moreno P, Freitas Fernandes F, Oliveira Brito PKM, Martinez R, Coelho C, Roque-Barreira MC, Casadevall A, Almeida F. 2019. Galectin-3 inhibits Paracoccidioides brasiliensis growth and impacts paracoccidioidomycosis through multiple mechanisms. mSphere 4:1–10. doi:10.1128/mSphere.00209-19 PMC6483048 31019001

[89] Zajta E, Csonka K, Tóth A, Tiszlavicz L, Németh T, Orosz A, Novák Á, Csikós M, Vágvölgyi C, Mócsai A, Gácser A. 2021. Signaling through Syk or CARD9 mediates species-specific anti-Candida protection in bone marrow chimeric mice. mBio 12:e0160821. doi:10.1128/mBio.01608-21 34465030 PMC8406149

[90] Bi L, Gojestani S, Wu W, Hsu YMS, Zhu J, Ariizumi K, Lin X. 2010. CARD9 mediates dectin-2-induced IκBα kinase ubiquitination leading to activation of NF-kappaB in response to stimulation by the hyphal form of Candida albicans. J Biol Chem 285:25969–25977. doi:10.1074/jbc.M110.131300 20538615 PMC2923990

[91] Gu Y, Jia XM. 2023. STING negatively regulates antifungal immunity. Trends Microbiol 31:1090–1092. doi:10.1016/j.tim.2023.09.003 37741789

[92] Chen T, Feng Y, Sun W, Zhao G, Wu H, Cheng X, Zhao F, Zhang L, Zheng Y, Zhan P, Zhao W, Liu B, Gao C. 2023. The nucleotide receptor STING translocates to the phagosomes to negatively regulate anti-fungal immunity. Immunity 56:1727–1742. doi:10.1016/j.immuni.2023.06.002 37379835

[93] Brown Harding H, Kwaku GN, Reardon CM, Khan NS, Zamith-Miranda D, Zarnowski R, Tam JM, Bohaen CK, Richey L, Mosallanejad K, Crossen AJ, Reedy JL, Ward RA, Vargas-Blanco DA, Basham KJ, Bhattacharyya RP, Nett JE, Mansour MK, van de Veerdonk FL, Kumar V, Kagan JC, Andes DR, Nosanchuk JD, Vyas JM. 2024. Candida albicans extracellular vesicles trigger type I IFN signalling via cGAS and STING. Nat Microbiol 9:95–107. doi:10.1038/s41564-023-01546-0 38168615 PMC10959075

[94] Freitas MS, Pessoni AM, Coelho C, Bonato VLD, Rodrigues ML, Casadevall A, Almeida F. 2021. Interactions of extracellular vesicles from pathogenic fungi with innate leukocytes, p 89–120. *In* Current topics in microbiology and immunology 10.1007/978-3-030-83391-6_9 34972881

[95] Peng M, Li X, Zhang X, Peng L. 2023. Inhibition of cGAS aggravated the host inflammatory response to Aspergillus fumigatus Exp Lung Res 49:86–100. doi:10.1080/01902148.2023.2211663 37190937

[96] Kulig K, Karnas E, Woznicka O, Kuleta P, Zuba-Surma E, Pyza E, Osyczka A, Kozik A, Rapala-Kozik M, Karkowska-Kuleta J. 2022. Insight into the properties and immunoregulatory effect of extracellular vesicles produced by Candida glabrata, Candida parapsilosis, and Candida tropicalis biofilms. Front Cell Infect Microbiol 12:879237. doi:10.3389/fcimb.2022.879237 35734578 PMC9207348

[97] Liu J, Hu X. 2023. Fungal extracellular vesicle-mediated regulation: from virulence factor to clinical application. Front Microbiol 14. doi:10.3389/fmicb.2023.1205477 PMC10540631 37779707

[98] Rizzo J, Trottier A, Moyrand F, Coppée J-Y, Maufrais C, Zimbres ACG, Dang TTV, Alanio A, Desnos-Ollivier M, Mouyna I, Péhau-Arnaude G, Commere P-H, Novault S, Ene IV, Nimrichter L, Rodrigues ML, Janbon G. 2023. Coregulation of extracellular vesicle production and fluconazole susceptibility in Cryptococcus neoformans. mBio 14:e0087023. doi:10.1128/mbio.00870-23 37310732 PMC10470540

[99] Lopes W, Vainstein MH, De Sousa Araujo GR, Frases S, Staats CC, de Almeida RMC, Schrank A, Kmetzsch L, Vainstein MH. 2017. Geometrical distribution of Cryptococcus neoformans mediates flower-like biofilm development. Front Microbiol 8:2534. doi:10.3389/fmicb.2017.02534 29312225 PMC5742216

[100] Livak KJ, Schmittgen TD. 2001. Analysis of relative gene expression data using real-time quantitative PCR and the 2(-delta delta C(T)) method. Methods 25:402–408. doi:10.1006/meth.2001.1262 11846609

