# Supplementary material for: Fungal extracellular vesicles mediate conserved cross-species communication and immunomodulation
Source: mBio. 2026 Jan 30;17(3):e03469-25. doi: 10.1128/mbio.03469-25 (PMC12977587; doi:10.1128/mbio.03469-25)
Supplement: Supplemental material — Supplemental figures and table. [file mbio.03469-25-s0001.docx]

**Supplementary Information**

**Fungal Extracellular Vesicles Mediate Conserved Cross-Species**

**Communication and Immunomodulation**

Renan E. A. Piraine^1^, Julia L. Froldi^1^, Henrique T. Oliveira^1^, Patrick W. Santos^1^, Bianca T. M. Oliveira^1^, Caroline P. Rezende^2^, Lucas Alves Tavares^3^, Gabriel E. S. Trentin^1^, Lucas F. B. Nogueira^1^, Arnaldo Colombo^4,5,6^, Arturo Casadevall^7^, Marcio L. Rodrigues^6,8,9^, Fausto Almeida^1,6^

**Results**

**Fungal EVs as messengers in intra- and inter-species communication**

**
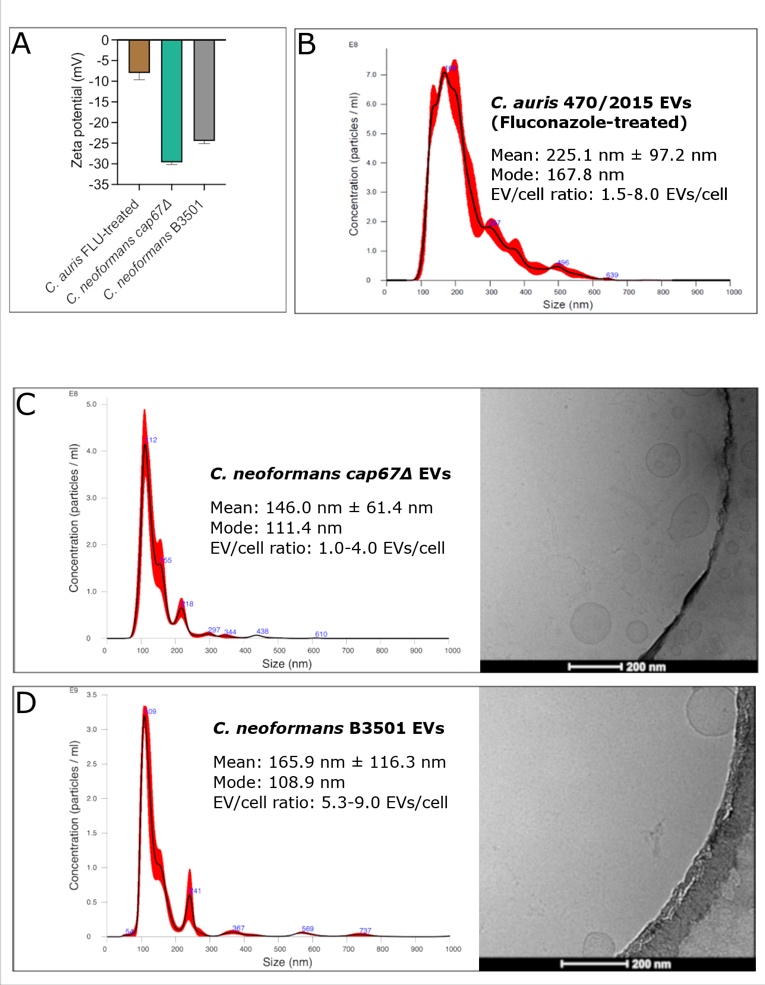
**

**Fig S1. Zeta potential, NTA, and Cryo-EM of additional fungal EVs used in this study.** Characterization of extracellular vesicles isolated from cultures of *C. auris* 470/2015 treated with fluconazole (A and B), *C. neoformans cap67*Δ acapsular strain (A and C) and *C. neoformans* B3501 (wild-type, reference strain *C. neoformans* var. *neoformans*) (A and D).

**
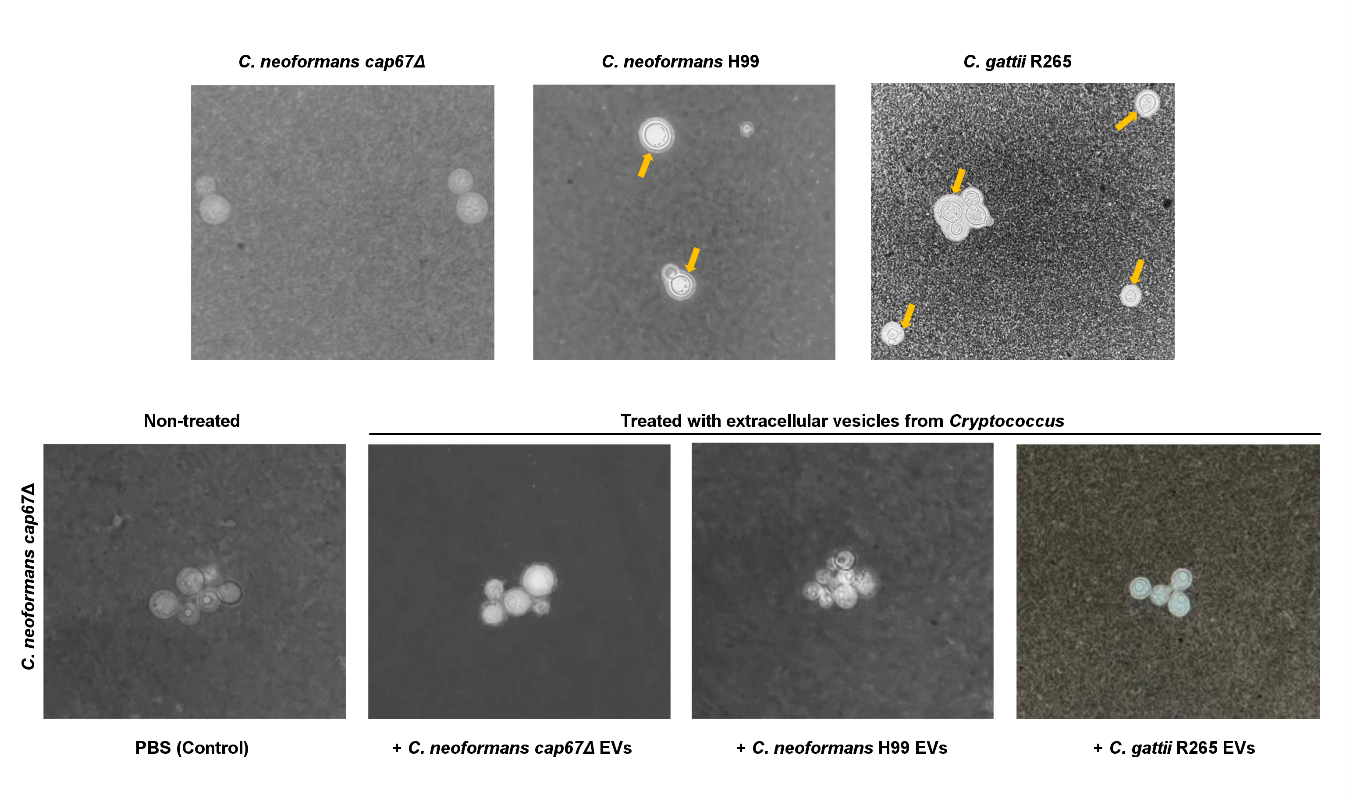
**

**Fig S2. India ink staining of the acapsular mutant *C. neoformans cap67*Δ and wild-type controls.** Wild-type encapsulated strains *C. neoformans* H99 and *C. gattii* R265 (upper side), and non-treated *C. neoformans cap67*Δ (PBS added), *C. neoformans cap67*Δ + EVs *C. neoformans cap67*Δ, *C. neoformans cap67*Δ + EVs *C. neoformans* H99, and EV*C. neoformans cap67*Δ + EVs *C. gattii* R265 (bottom side). Images of India ink-stained samples were obtained under light microscope at 100x magnification. Yellow arrows indicate the polysaccharide capsule present at the surface of wild-type strains.


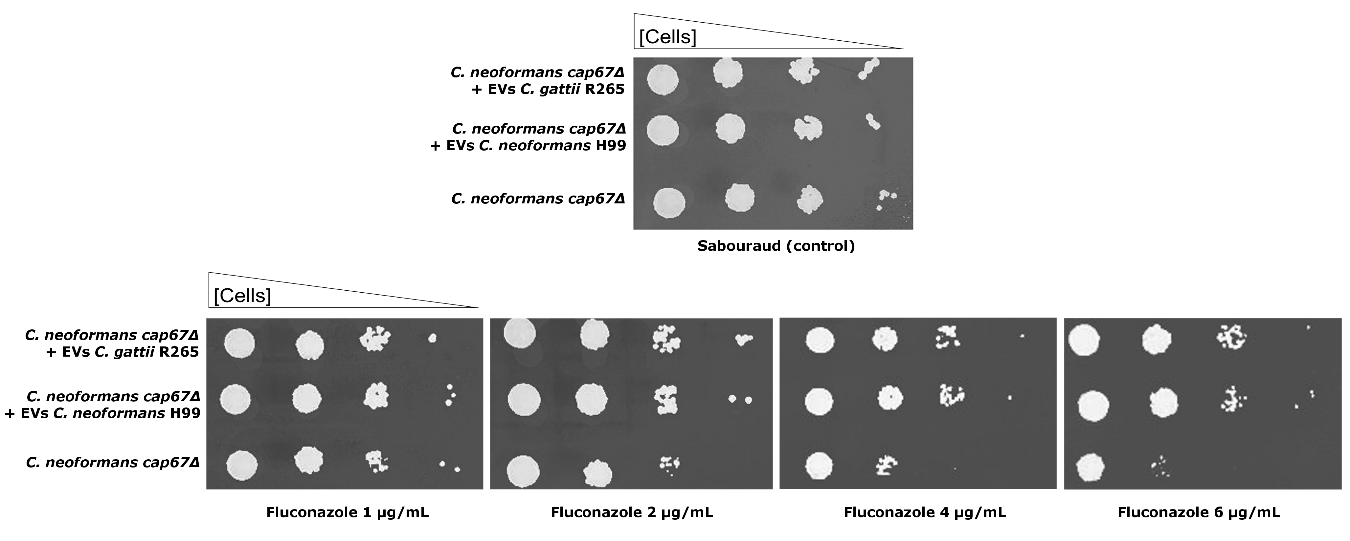


**Fig S3. Spot test of *C. neoformans cap67*Δ treated with EVs from *C. neoformans* and *C. gattii* and incubated in different concentrations of fluconazole.** The impact in fluconazole tolerance of *C. neoformans cap67*Δ after EVs addition, both in intraspecies and interspecies interactions, was evaluated using a spot test in Sabouraud agar supplemented with crescent dilutions (1 μg/mL to 6 μg/mL) of the antifungal. Samples of cultures were normalized at 10^7^ cells/mL, washed three times with PBS, and diluted until 10^4^ with PBS, then 10 μL were applied for each sample. Plates were incubated under 37 °C for 72 h.

**Material and Methods**

**Table S1. Sequences of primers used in this study for quantitative real-time PCR (qPCR)**

| **Gene** | **Sense** | **Sequence (5’ →3’)** | **Source** |
| --- | --- | --- | --- |
| ***Human*** |  |  |  |
| *ACT1*  (β-Actin) | Forward  Reverse | ATTGCCGACAGGATGCAGAA  GCTGATCCACATCTGCTGGAA | (1) |
| *iNOS*  (Inducible nitric oxide synthase) | Forward  Reverse | CATCCTCTTTGCGACAGAGAC  GCAGCTCAGCCTGTACTTATC | (2) |
| *ARG1*  (Arginase 1) | Forward  Reverse | GCCCTTTGCTGACATCCCTA  CGCTTGCTTTTCCCACAGAC | (3) |
| *TLR2*  (Toll-like receptor 2) | Forward  Reverse | CAGGAGCTCTTAGTGACCAAGTGAA  CACAAAGTATGTGGCATTGTCCAG | (4) |
| *TLR4*  (Toll-like receptor 4) | Forward  Reverse | GGTCAGACGGTGATAGCGAG  TTTAGGGCCAAGTCTCCACG | (2) |
| *TLR9*  (Toll-like receptor 9) | Forward  Reverse | TCTTGAAGGCCTGGTGTTGA  AAGGACAGGTTAAGCTTGCG | (5) |
| *CLEC7A*  (C-type lectin domain containing 7A – Dectin1) | Forward  Reverse | AGCCTACCTGTAGGTCGACAA  CTGAGGTCAAGATAAATGCAGAAA | (6) |
| *LGALS3*  (Lectin galactose binding soluble 3 - Galectin3) | Forward  Reverse | TATCCTGCTGCTGGCCCTTAT  CACTGTGCCCATGATTGTGATC | (7) |
| *CGAS*  (Cyclic- GMP-AMP synthase) | Forward  Reverse | CAAGAAGGCCTGCGCATTCA  GAGAAGGATAGCCGCCATGT | (8) |
| *STING*  (Stimulator of interferon genes) | Forward  Reverse | GATATCTGCGGCTGATCCTG  GCTGTAAACCCGATCCTTGA | (8) |
| *SYK*  (Spleen associated tyrosine kinase) | Forward  Reverse | CATGGAAAAATCTCTCGGGAAGA  GTCGATGCGATAGTGCAGCA | (9) |
| *CARD9*  (Caspase recruitment domain family member 9) | Forward  Reverse | TCCGACCTGGAAGATGGCTCAC  CAGAGCTGCAAAGGGCTGTTTC | (10) |
| *MYD88*  (Myeloid differentiation primary response gene 88) | Forward  Reverse | GCATATGCCTGAGCGTTTCG  TTCTGATGGGCACCTGGAGA | (11) |
| ***Fungus*** |  |  |  |
| *18s rRNA*  (18s ribosomal RNA) | Forward  Reverse | CCGTTGCTAGAGGTGAAATTCTTAG  ATCTAATCGTTTTTGATCCCCTAAC | (12) |
| *ERG11*  (Sterol 14α-demethylase) | Forward  Reverse | ACCCTGAAGATTTTGATCCAACTAGATG  CCCAAACCCATAATCAATTCATCAGA | (13) |
| *LAC1*  (Laccase 1) | Forward  Reverse | AGAAGGGAAGGAAGGTGATG  TATACCTCACAACCGCCAAT | (12) |
| *URE1*  (Urease) | Forward  Reverse | TCGTATCGGTGAAGTCGTCACT  GGACCACGGAATTGCTTCAT | (14) |
| *CAP59* | Forward  Reverse | GATCTGGGAGCGCTGTAGAC  TCTCTGCTCGTCGGTATCCT | (14) |

**References**

1. Maeß MB, Sendelbach S, Lorkowski S. 2010. Selection of reliable reference genes during THP-1 monocyte differentiation into macrophages. *BMC Mol Biol* 11:90.

2. Bisht A, Dickens M, Rutherfurd-Markwick K, Thota R, Mutukumira AN, Singh H. 2020. Chlorogenic acid potentiates the anti-inflammatory activity of curcumin in LPS-stimulated THP-1 cells. *Nutrients* 12:1–12.

3. Pritchard A, Tousif S, Wang Y, Hough K, Khan S, Strenkowski J, Chacko BK, Darley-Usmar VM, Deshane JS. 2020. Lung Tumor Cell-Derived Exosomes Promote M2 Macrophage Polarization. *Cells* 9.

4. Li M, Li X, Wang E, Luo E. 2013. Upregulation of Toll-like receptor 2 gene expression by acetylation ofAP-2 alpha in THP-1 cells, a human monocytic cell line. *Int J Biochem* *Cell Biol* 45:1594–1599.

5. Trinh TA, Hoang TX, Kim JY. 2020. All-trans retinoic acid increases NF-κB activity in PMA-stimulated THP-1 cells upon unmethylated CpG challenge by enhancing cell surface TLR9 expression. *Mol Cell Biochem* 473:167–177.

6. Peng Y, Chen YAN, Ma J, Zhou WEI, Wang Y, Wang Y, Zheng HUI, Shi W. 2022. Role and mechanism of the Dectin ‑ 1 ‑ mediated Syk / NF ‑ κ B signaling pathway in *Talaromyces marneffei* infection. *Exp Ther Med* 1–9.

7. Esteban A, Popp MW, Vyas VK, Strijbis K, Ploegh HL, Fink GR. 2011. Fungal recognition is mediated by the association of dectin-1 and galectin-3 in macrophages. *Proc Natl Acad Sci* U S A 108:14270–14275.

8. Lauterbach-Rivière L, Bergez M, Mönch S, Qu B, Riess M, Vondran FWR, Liese J, Hornung V, Urban S, König R. 2020. Hepatitis B virus DNA is a substrate for the cGAS/STING pathway but is not sensed in infected hepatocytes. *Viruses* 12.

9. Chen J, Jia G, Lv X, Li S. 2022. Type 1 Diabetes Mellitus-Related circRNAs Regulate CD4+ T Cell Functions. *Biomed Res* Int 2022.

10. Mao L, Zhang L, Li H, Chen W, Wang H, Wu S, Guo C, Lu A, Yang G, An L, Abliz P, Meng G. 2014. Pathogenic fungus *Microsporum canis* activates the NLRP3 inflammasome. *Infect Immun* 82:882–892.

11. Cirmi S, Maugeri A, Russo C, Musumeci L, Navarra M, Lombardo GE. 2022. Oleacein Attenuates Lipopolysaccharide‐Induced Inflammation in THP‐1‐Derived Macrophages by the Inhibition of TLR4/MyD88/NF‐κB Pathway. *Int J Mol Sci* 23.

12. García-Rodas R, Trevijano-Contador N, Román E, Janbon G, Moyrand F, Pla J, Casadevall A, Zaragoza O. 2015. Role of Cln1 during melanization of *Cryptococcus neoformans*. *Front Microbiol* 6:1–9.

13. Zare-Bidaki M, Maleki A, Ghanbarzadeh N, Nikoomanesh F. 2022. Expression pattern of drug-resistance genes ERG11 and TAC1 in *Candida albicans* Clinical isolates. *Mol Biol Rep* 49:11625–11633.

14. Benaducci T, Sardi J de CO, Lourencetti NMS, Scorzoni L, Gullo FP, Rossi SA, Derissi JB, de Azevedo Prata MC, Fusco-Almeida AM, Mendes-Giannini MJS. 2016. Virulence of *Cryptococcus* sp. biofilms in vitro and in vivo using *Galleria* *mellonella* as an alternative model. *Front Microbiol* 7:1–10.
